# Homologous *Tremella fuciformis* semi-IPN hydrogels with entanglement-assisted network stabilization for near-neutral pH-responsive photodamage therapy

**DOI:** 10.1016/j.mtbio.2026.103503

**Published:** 2026-08-04

**Authors:** Fei Lin, Huimin Xie, Fang Luo, Zhenyu Lin, Yong Wang, Bin Qiu, Yanjing Huang

**Affiliations:** a Ministry of Education Key Laboratory for Analytical Science of Food Safety and Biology, Fujian Provincial Key Laboratory of Analysis and Detection for Food Safety, Fuzhou University, Fuzhou, Fujian, 350108, China; b Department of Applied Biology and Chemical Technology, The Hong Kong Polytechnic University, Hung Hom, Kowloon, Hong Kong Special Administrative Region of China; c Department of Emergency Center, The First Affiliated Hospital, Fujian Medical University, Fuzhou, 350005, China; d Department of Emergency, National Regional Medical Center, Binhai Campus of the First Affiliated Hospital, Fujian Medical University, Fuzhou, 350212, China

**Keywords:** *Tremella fuciformis* polysaccharides, Homologous semi-interpenetrating polymer network, Near-neutral pH-responsiveness, Transdermal drug delivery, Photodamage therapy, Quercetin

## Abstract

Healthy skin maintains a weakly acidic surface pH (4.1-5.8), whereas barrier disruption and acute inflammation may shift the local microenvironment toward near-neutral conditions. However, most stimuli-responsive hydrogels are designed for acidic environments, limiting their suitability for photodamaged skin. To address this, we developed a homologous semi-interpenetrating polymer network (semi-IPN) hydrogel by integrating native *Tremella fuciformis polysaccharides* (TFPs), oxidized TFPs (OTFPs), and carboxymethyl chitosan (CMCS) for near-neutral pH-responsive transdermal delivery of quercetin (QT). Native TFPs introduced long-chain physical chain entanglement, improving network stability, flexibility (96.6% strain), and water retention. 10 wt% CMCS increased the apparent solubility of QT to 2409.8 μg/mL and facilitated homogeneous drug dispersion. Benefiting from the high hydration capacity of TFPs, the system enhanced transdermal delivery, achieving a 6.2-fold increase in permeability compared with glyoxal (GA)-crosslinked hydrogels. The semi-IPN hydrogel showed near-neutral pH-responsive swelling behavior, increasing from 2829% at pH 4.5 to 4275% at pH 7.4, which triggered 59.7% QT release within 24 h. *In vitro*, the hydrogel restored HaCaT cell viability to 90.5% and reduced reactive oxygen species (ROS) by 76.9% after UVB exposure. *In vivo*, it accelerated structural repair, reducing epidermal thickness from 202.5 μm to 54.6 μm and increasing collagen content to 41.4%, while also providing photoprotection. This work presents a near-neutral pH-responsive homologous polysaccharide hydrogel for transdermal photodamage therapy and protection.

## Highlights

• Homologous semi-IPN improves network flexibility and hydration capacity.
• Carboxymethyl chitosan significantly enhances quercetin solubility and loading.
• Near-neutral pH-responsive swelling accelerates drug release under skin damage conditions.
• Hydrated polysaccharide networks enhance quercetin transdermal permeability by 6.2-fold.
• Quercetin-loaded TOC reduces ROS by 76.9% and promotes UV protection and skin repair.

## Graphical abstract

Scheme 1. Entanglement-Stabilized Homologous *Tremella fuciformis* Polysaccharide Semi-IPN Hydrogels for Near-Neutral pH-Responsive Photodamage Therapy.

**Figure:**
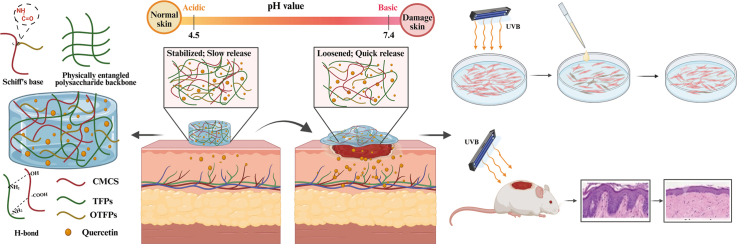

## 1 Introduction

Healthy human skin maintains a weakly acidic surface pH (4.1-5.8), known as the acid mantle, which is critical for barrier homeostasis and antimicrobial defense [[1], [2], [3], [4]]. UVB irradiation and other environmental stressors can disrupt stratum corneum integrity, leading to barrier dysfunction and inflammatory responses [[5], [6], [7]]. Skin barrier impairment has been reported to shift the local microenvironment toward neutral or weakly alkaline conditions, a pathological pH alteration also observed in acute wounds and various dermatitis conditions [[8], [9], [10], [11], [12], [13], [14]]. These findings suggest that a barrier-associated near-neutral microenvironment may serve as a physiological cue for designing pH-responsive biomaterials for photodamaged skin treatment.

Stimuli-responsive hydrogels, particularly pH-responsive systems, serve as versatile platforms for wound repair and local drug delivery due to their ability to dynamically regulate network structures [[15], [16], [17], [18]]. Recently, natural polysaccharide matrices, including alginate, hyaluronic acid, and chitosan derivatives, have been widely investigated for skin repair and transdermal delivery applications [19,20]. The responsive behaviors of these systems are mainly regulated by functional group protonation/deprotonation, electrostatic interactions, and dynamic covalent bonds. As demonstrated by sodium alginate/carboxymethyl chitosan complexes, these molecular changes can promote pH-responsive network adjustment and controlled payload release through reversible hydrogen bonding and ionic interactions [21]. Most reported pH-responsive hydrogels are designed for acidic pathological environments, such as tumors or infected wounds, and may not fully match the near-neutral microenvironment associated with skin barrier impairment [14,22]. Balancing mechanical strength and biological performance remains challenging, as reinforcing mechanical properties through synthetic polymer backbones or dense crosslinking often compromises gel flexibility, water retention, and biocompatibility [23]. Developing natural polysaccharide-based hydrogels that integrate near-neutral pH responsiveness, network stability, and efficient transdermal delivery remains a major challenge.

Among natural polysaccharides, *Tremella fuciformis* polysaccharides (TFPs) are considered promising skin biomaterials due to their high molecular weight, hyperbranched structure, and excellent moisture retention properties [[24], [25], [26]]. Structurally, the TFP backbone is rich in mannose, while glucuronic acid residues in the side chains provide ionizable sites that contribute to pH responsiveness [26,27]. The high molecular weight and limited reactive groups of native TFPs restrict further functionalization and crosslinking, hindering the construction of structurally controllable and dynamically responsive hydrogels [[28], [29], [30]]. Chemical oxidation is commonly used to introduce aldehyde groups and increase carboxyl content, thereby improving reactivity and environmental responsiveness [31,32]. However, oxidation is often accompanied by severe chain scission and molecular weight reduction, which can compromise network continuity, mechanical stability, and water retention capacity [31,33]. Balancing functional modification and structural preservation remains a key challenge in the development of TFP-based responsive hydrogels.

Previously, we developed self-healing hydrogels by crosslinking oxidized *Tremella fuciformis* polysaccharides (OTFPs) with carboxymethyl chitosan (CMCS) via dynamic Schiff base bonds, demonstrating promising antioxidant and skin-repair activities [31]. The oxidation process inherently caused extensive molecular degradation, resulting in a substantial reduction in the molecular weight of OTFPs. Because the entanglement of long polymer chains contributes to network continuity, water retention, and mechanical stability under highly hydrated conditions [[34], [35], [36]], the loss of these long chains may reduce physical entanglement and compromise hydrogel structural integrity [37,38]. The present study introduces native high-molecular-weight TFPs into the OTFPs/CMCS matrix to construct a homologous semi-interpenetrating polymer network (semi-IPN). This architecture utilizes the physical entanglement of native TFP chains to enhance network stability while preserving the dynamic crosslinking and environmental responsiveness of the OTFPs component.

Besides optimizing the hydrogel matrix, selecting an effective therapeutic agent is also essential for treating UVB-induced skin damage. Quercetin (QT) has attracted considerable attention for mitigating UVB-induced skin damage due to its multifunctional antioxidant, anti-inflammatory, and photoprotective activities [[39], [40], [41]]. Distinct from conventional antioxidants that mainly rely on radical scavenging, QT also exhibits UV absorption properties, enabling simultaneous photoprotection and skin damage repair [42,43]. Its topical application remains challenging due to poor aqueous solubility, molecular aggregation, and limited transdermal bioavailability [44,45]. This study incorporates CMCS as a functional matrix to reduce QT aggregation and enhance its apparent solubility through hydrogen bonding and other weak intermolecular interactions [46,47]. Beyond solubilization, the hydrophilic and biocompatible properties of CMCS contribute to maintaining a stable local delivery environment. Incorporating this system into a near-neutral pH-responsive hydrogel carrier that integrates drug solubilization, controlled release, and improved skin delivery provides an effective strategy to enhance the therapeutic potential of QT.

Guided by this rationale, we constructed a homologous semi-IPN hydrogel by integrating native TFPs, OTFPs, and CMCS for the microenvironment-responsive delivery of QT. We investigated how the introduction of intact native TFPs chains restores network continuity, water retention, and mechanical stability within the OTFPs/CMCS matrix. Furthermore, the hydrogel's near-neutral pH-responsive behavior, transdermal delivery characteristics, and therapeutic effects against UVB-induced photodamage were systematically evaluated. This work demonstrates a strategy for developing natural polysaccharide-based smart biomaterials with improved structural stability and microenvironment-responsive functions for skin repair.

## 2 Results

### 2.1 Characterization of OTFPs and TFPs

The key physicochemical parameters of native TFPs and OTFPs are summarized in Table 1. After oxidation, the successful introduction of reactive groups was confirmed by the significant increase in aldehyde content from 0.00% to 0.78% ± 0.03% and carboxyl content from 1.17% ± 0.05% to 8.93% ± 0.50%. Concurrently, a significant decrease in the weight average molecular weight was observed due to oxidative chain scission.

**Table 1:** Characterization of TFPs and OTFPs.

Precursor	Aldehyde content (%)	Carboxyl content (%)	Molecular weight (*Mw*, kDa)
**OTFPs**	0.78 ± 0.03	8.93 ± 0.50	5.00
**TFPs**	0.00	1.17 ± 0.05	1079.60

### 2.2 Preparation and characterization of hydrogels

Four hydrogel formulations were prepared, with their abbreviations, compositions, and gelation times (all ≤ 3 min) summarized in Table 2. Incorporation of TFPs increased precursor viscosity and slightly delayed gelation by restricting functional group mobility and slowing Schiff-base crosslinking [48].

**Table 2:** Summary of abbreviations, compositions, and gelation times for different hydrogel formulations.

Hydrogels	OTFPs (w/v)	CMCS (w/v)	TFPs (w/v)	Glyoxal (w/v)	QT (w/v)	Gelation Time (min)
**Glyoxal/CMCS (GC)**	-	9%	-	1%	-	1
**OTFPs/CMCS (OC)**	5%	5%	-	-	-	2
**TFPs/OTFPs/CMCS (TOC)**	5%	4%	1%	-	-	3
**TFPs/OTFPs/CMCS-QT (TOC-QT)**	5%	4%	1%	-	1%	1.5

**Fig. 1:**
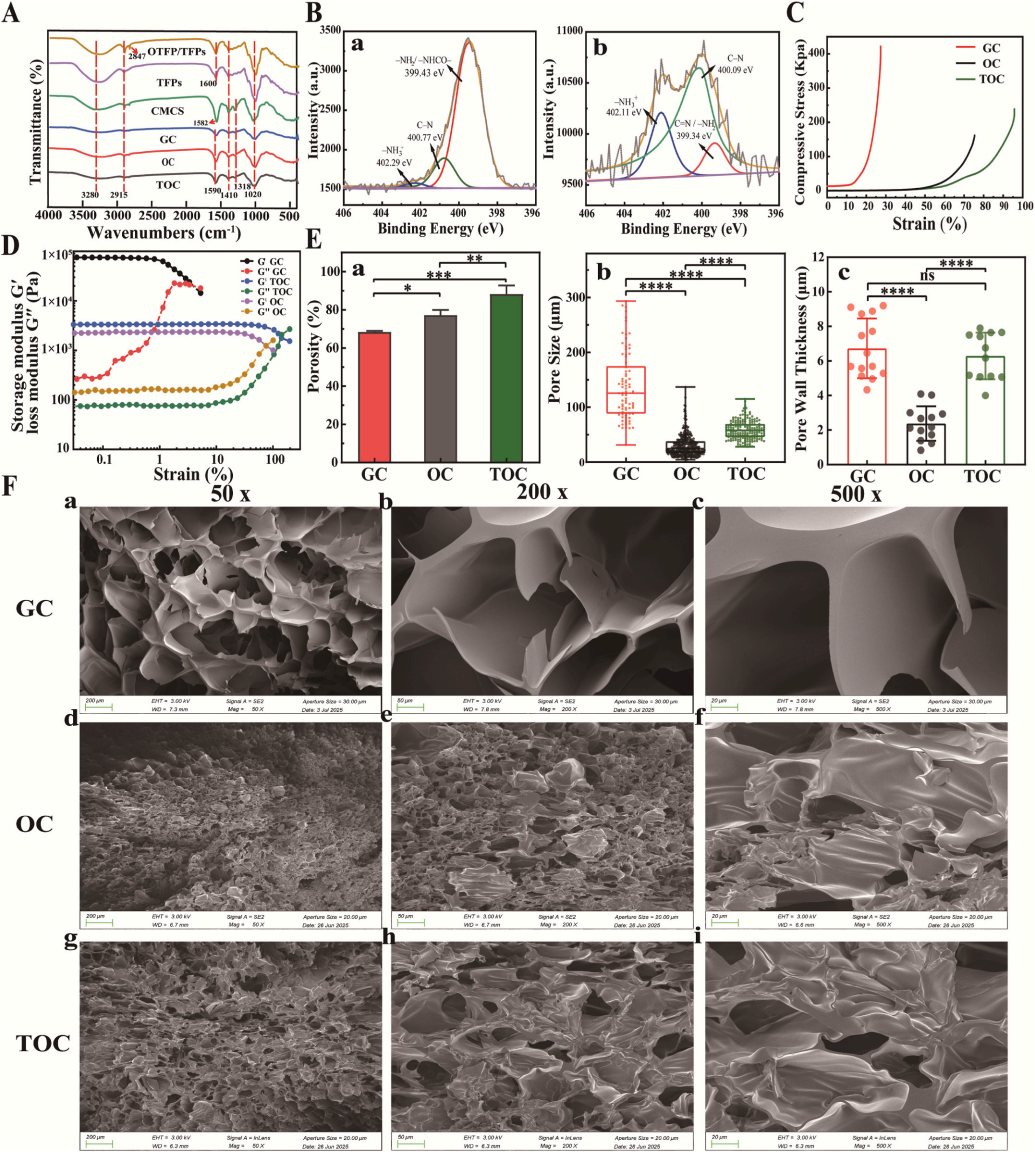
Structural characterization and physicochemical properties of the hydrogels. (A) FTIR spectra of the precursors (TFPs/OTFPs, TFPs, and CMCS) and the corresponding hydrogels (GC, OC, and TOC). (B) High-resolution XPS N1s spectra of (a) CMCS and (b) TOC hydrogels. (C) Compressive stress–strain curves of the hydrogels. (D) Oscillatory rheology (strain sweep) of the hydrogels. (E) Structural properties quantified from SEM images: (a) porosity, (b) average pore size, and (c) pore wall thickness. (F) SEM morphology of the hydrogels: (a–c) GC, (d–f) OC, and (g–i) TOC. Data are presented as mean ± SD (n ≥ 3; *p < 0.05, **p < 0.01, ***p < 0.001, ****p < 0.0001).

FTIR spectra revealed chemical changes during hydrogel formation (Fig. 1A). An absorption band near 2847 cm^−1^ appeared only in the TFPs/OTFPs sample, indicating oxidation-induced structural modification of OTFPs. Upon gelation, the broad O–H/N–H stretching region around 3280 cm^−1^ exhibited subtle profile changes, suggesting rearrangement of intermolecular hydrogen-bonding interactions. Concurrently, the characteristic COO^−^ band of CMCS (1582 cm^−1^) and the adjacent precursor band (∼1600 cm^−1^) merged and shifted to ∼1590 cm^−1^, indicating changes in the local chemical environment associated with gel formation. Meanwhile, the peaks at 2915 and 1020 cm^−1^ remained largely unchanged, suggesting preservation of the primary polysaccharide backbone during crosslinking.

Since CMCS serves as the sole nitrogen source, high-resolution N 1s XPS spectra were evaluated to probe changes in nitrogen chemical states after gelation (Fig. 1B), with the corresponding survey spectra provided in Fig. S11. Compared with pristine CMCS, the TOC hydrogel displayed a redistribution of nitrogen states. The deconvoluted peak at 399.34 eV was assigned to overlapping C=N/–NH_2_ species, while the remaining peaks at 400.09 and 402.11 eV corresponded to C–N bonds and protonated nitrogen, respectively. The altered relative contributions of these nitrogen species suggest changes in the nitrogen chemical environment and are consistent with partial amino-group participation during gelation. Taken together, FTIR and XPS analyses provide complementary evidence for changes in the chemical environment associated with Schiff-base crosslinking and hydrogel network formation.

### 2.3 Microstructure and physical properties of hydrogels

The hydrogels exhibited distinct microstructural features, as revealed by SEM images and quantitative data (Fig. 1E, F and Fig. S2). TFPs primarily formed physically entangled structures with large, irregular pores and thin walls, while the TFPs/OTFPs samples showed more uniform pores and smoother surfaces (Fig. S2). Covalently crosslinked GC possessed the lowest porosity (68.4%), a large average pore size (137.6 ± 62.2 μm), and thick pore walls (6.7 μm), indicative of an irregular and partially collapsed structure. In comparison, OC displayed a refined pore structure and an increased porosity of 77.2% after covalent crosslinking via OTFPs. The TOC semi-IPN combined chemical crosslinks with physical entanglements to form a highly interconnected and uniform porous network, achieving the highest porosity (88.2%) and a well-regulated average pore size of 58.5 ± 17.1 μm. Its wrinkled pore walls may provide additional deformation space and facilitate local stress redistribution, thereby contributing to enhanced energy dissipation and mechanical resilience [49,50].

Water evaporation and contact angle measurements highlighted the functional impacts of these microstructural differences (Figs. S3 and S4). GC exhibited the largest water contact angle (50.8°), indicating a relatively hydrophobic surface, whereas OC and TOC showed enhanced surface hydrophilicity with smaller contact angles of 32.7° and 26.3°, respectively. Correspondingly, after 8 h of evaporation, cumulative water loss was highest for OC (60.5%) and lowest for GC (45.4%). Interestingly, despite possessing the highest surface hydrophilicity, TOC lost less water than OC (51.4%). This confirms that moisture retention was governed not merely by surface wettability, but was heavily influenced by the internal network architecture [51], where the interconnected semi-IPN channels effectively restricted water migration and vapor diffusion.

Mechanical and rheological profiles further substantiated these structure–property relationships (Fig. 1C and D). GC was stiff but brittle, possessing a compressive strength of 367.7 ± 40.8 kPa and a low fracture strain of 32.2 ± 6.5%. OC was more extensible (74.7 ± 2.2% strain) but weaker (161.2 ± 11.0 kPa). In contrast, the TOC semi-IPN successfully balanced these metrics, exhibiting both high extensibility (96.6 ± 1.5% strain) and moderate-to-high stress tolerance (235.7 ± 25.3 kPa). Rheologically, GC displayed a storage modulus (*G′*) significantly higher than its loss modulus (*G″*) at low strains, followed by a sharp drop under higher strains due to the limited stress-relaxation capability of its rigid covalent network. OC showed a narrower gap between *G′* and *G″*, indicating a larger viscous contribution. In the TOC semi-IPN, reversible physical entanglements and hydrogen-bonding interactions permitted efficient chain sliding [52] and rearrangement under shear stress, expanding the linear viscoelastic region and enhancing energy dissipation.

Overall, this semi-IPN architecture establishes a clear synergy between uniform microstructure, mechanical toughness, and moisture retention, successfully balancing robust structural support with high stretchability.

### 2.4 pH- and ionic-responsive swelling behavior

**Fig. 2:**
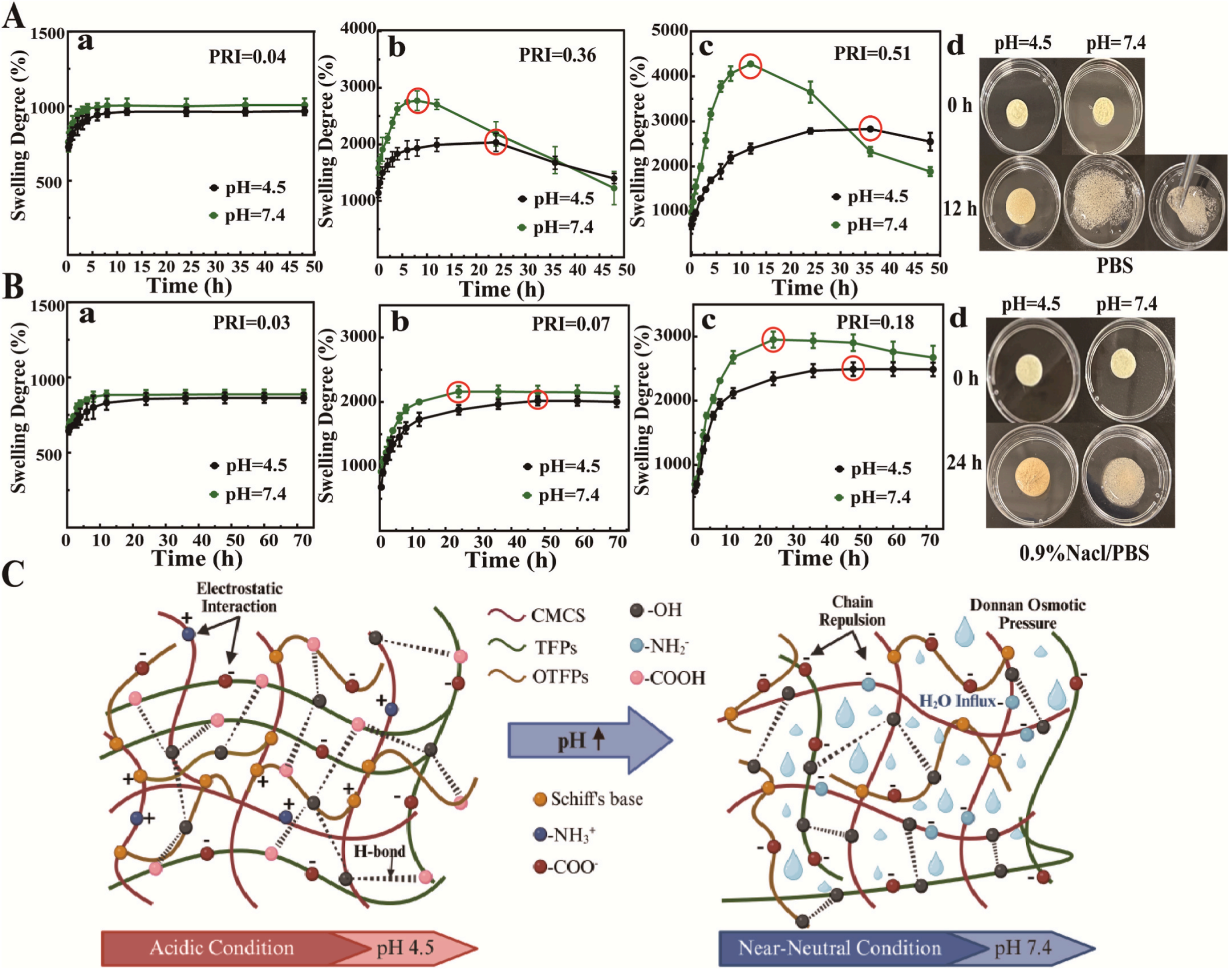
pH- and ionic-responsive swelling behaviors and proposed mechanism of the hydrogel matrices. (A) pH-responsive swelling behavior of hydrogels in PBS (37 °C, 48 h): (a) GC, (b) OC, (c) TOC, and (d) digital photographs of TOC hydrogels at 12 h. (B) Ionic strength-dependent swelling behavior of hydrogels in PBS containing 0.9% NaCl (37 °C, 72 h): (a) GC, (b) OC, (c) TOC, and (d) digital photographs of TOC hydrogels at 24 h. (C) Schematic illustration of the pH-responsive mechanism of the TOC semi-IPN hydrogel.

Healthy skin typically maintains a weakly acidic surface pH, whereas barrier-impaired skin may shift toward near-neutral conditions. To mimic these environments, swelling behavior was evaluated in PBS at pH 4.5 and 7.4. Among the three hydrogels, GC showed the lowest swelling at both pH values, likely due to its highly crosslinked and rigid network. In contrast, OC swelled quickly, reaching maximum swelling ratios of 2035% at pH 4.5 and 2771% at pH 7.4 (PRI = 0.36), but its swelling degree decreased at later times, suggesting limited long-term structural stability. TOC exhibited the most pronounced pH-responsive behavior, with maximum swelling increasing from 2829% at pH 4.5 to 4275% at pH 7.4 (PRI = 0.51) (Fig. 2A a–c). This marked swelling at pH 7.4 was also evident in the photographs taken at 12 h (Fig. 2A d), where the hydrogel expanded substantially while still retaining a coherent gel-like structure. SEM images (Fig. S6) further showed that TOC at pH 7.4 formed a looser network with larger pores, which likely allows more water uptake and facilitates faster transport within the hydrogel. To examine the influence of ionic strength, the hydrogels were also immersed in PBS containing 0.9% NaCl (Fig. 2B). In this environment, all hydrogels swelled less, and the PRI values decreased noticeably. For TOC, the maximum swelling at pH 7.4 dropped to 2952%, about 69% of the value in pure PBS, indicating that salt screening of network charges reduces the swelling response.The mechanism behind this pH-responsive swelling is summarized in Fig. 2C. At pH 4.5, electrostatic attraction between protonated amino groups (-NH_3_^+^) and partially dissociated carboxyl groups (-COO^-^) in CMCS, combined with dense hydrogen-bonding [53,54], likely restricts chain mobility and keeps the network compact. Raising the pH to 7.4 reduces these attractive interactions, while increased carboxyl deprotonation generates net negative charges. The resulting electrostatic repulsion drives water uptake and pore expansion [[55], [56], [57]], and rearrangement of hydrogen bonds may further relax the network, enhancing the swelling response.

(PRI: pH-responsive index, Data are presented as mean ± SD, n = 3.)

### 2.5 Loading of quercetin in semi-IPN hydrogel and hydrogel degradation

**Fig. 3:**
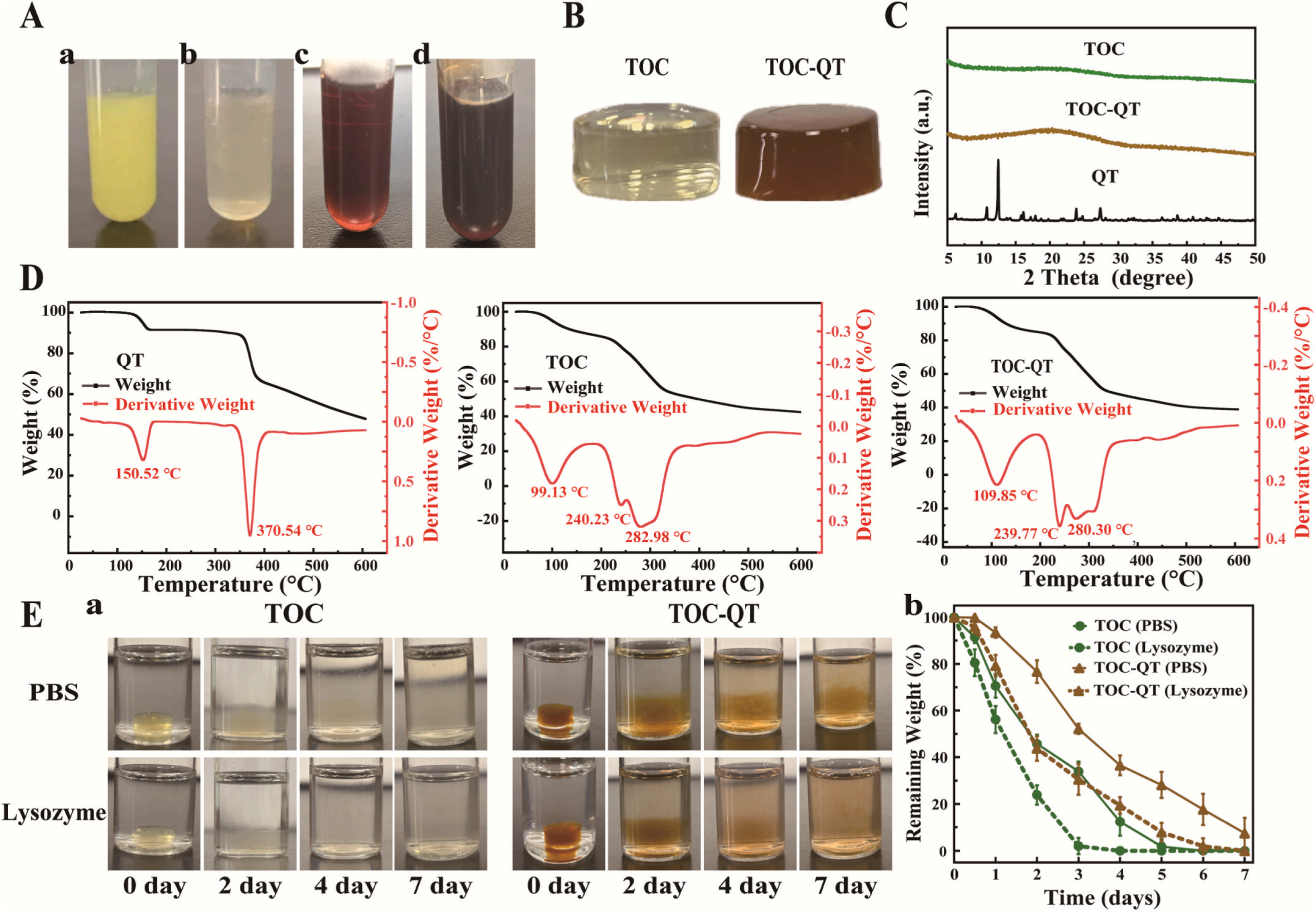
Drug loading and *in vitro* degradation of TOC hydrogels. (A) CMCS-mediated solubilization of QT: (a) 1.0 mg/mL QT in H_2_O, (b) 10% CMCS, (c) 1.0 mg/mL QT in 5% CMCS, and (d) 2.0 mg/mL QT in 10% CMCS. (B) Digital photographs of TOC and TOC-QT hydrogels. (C) XRD patterns of crystalline QT, TOC, and TOC-QT. (D) TG (black) and DTG (red) curves of QT, TOC, and TOC-QT. (E) *In vitro* degradation of TOC and TOC-QT in PBS and lysozyme: (a) morphological changes over 7 days, and (b) corresponding remaining weight curves (n = 3). (For interpretation of the references to colour in this figure legend, the reader is referred to the Web version of this article.)

Due to its hydrophobic nature, QT exhibits poor aqueous solubility, which limits its biological applications [58]. To address this limitation, CMCS was employed as a solubilizing matrix [46,47]. As shown in Fig. 3A, QT was successfully dispersed in CMCS solutions without visible precipitation, achieving concentration-dependent apparent solubilities of 1141.6 ± 72.6 μg/mL and 2409.8 ± 84.9 μg/mL in 5% and 10% (w/v) CMCS solutions, respectively. Upon incorporation into the hydrogel precursor, the system transitioned from light yellow to brownish-red (Fig. 3B), indicating successful drug entrapment within the polymer matrix. Given the enclosed fabrication process without washing or separation steps, material loss during gelation was negligible. Thus, drug loading was directly determined from the initial precursor concentration and mixing ratio.

The physical state of QT within the hydrogel was further analyzed by X-ray diffraction (Fig. 3C). The absence of characteristic crystalline peaks of pure QT in the hydrogel profile indicated a loss of long-range crystalline order, suggesting that QT existed in an amorphous or highly dispersed state within the polymer network. To further evaluate QT incorporation and the thermal behavior of the hydrogel, thermogravimetric analysis (TGA) was performed (Fig. 3D). The TOC hydrogel exhibited three characteristic degradation stages with DTG maxima at 99.13 °C, 240.23 °C, and 282.98 °C, corresponding to the loss of physically bound water, initial polysaccharide backbone cleavage, and main chain decomposition, respectively. After QT incorporation, the TOC-QT hydrogel showed similar degradation stages, with DTG peaks at 109.85 °C, 239.77 °C, and 280.30 °C. A slight shift in the dehydration peak and a more pronounced DTG signal near 240 °C suggest minor changes in water retention and local thermal behavior after QT incorporation, while the overall degradation pathway remained unchanged. These observations further support favorable incorporation of QT within the hydrogel network without major structural disruption.

Subsequently, the degradation behavior of the hydrogels was evaluated in PBS with or without 0.5 mg/mL lysozyme to assess their structural stability in wound-mimicking environments (Fig. 3E). As shown in the remaining weight curves (Fig. 3E b), lysozyme significantly accelerated hydrogel degradation, suggesting that the polysaccharide-based network possesses enzymatic susceptibility under biologically relevant conditions. The unloaded TOC hydrogel degraded almost completely by day 3 in the presence of lysozyme. Conversely, the TOC-QT hydrogel exhibited prolonged stability, retaining approximately 10% of its initial mass after 7 days under lysozyme-containing conditions. Macroscopic observations (Fig. 3E a) further supported this trend, showing delayed erosion of the drug-loaded formulation. This enhanced resistance indicates improved structural stability of the QT-loaded hydrogel during degradation. Furthermore, QT loading significantly enhanced the UV–Vis absorption of the semi-IPN hydrogel (Fig. S5), highlighting its potential for photoprotective applications.

### 2.6 *In vitro* drug release and kinetic mechanisms

**Fig. 4:**
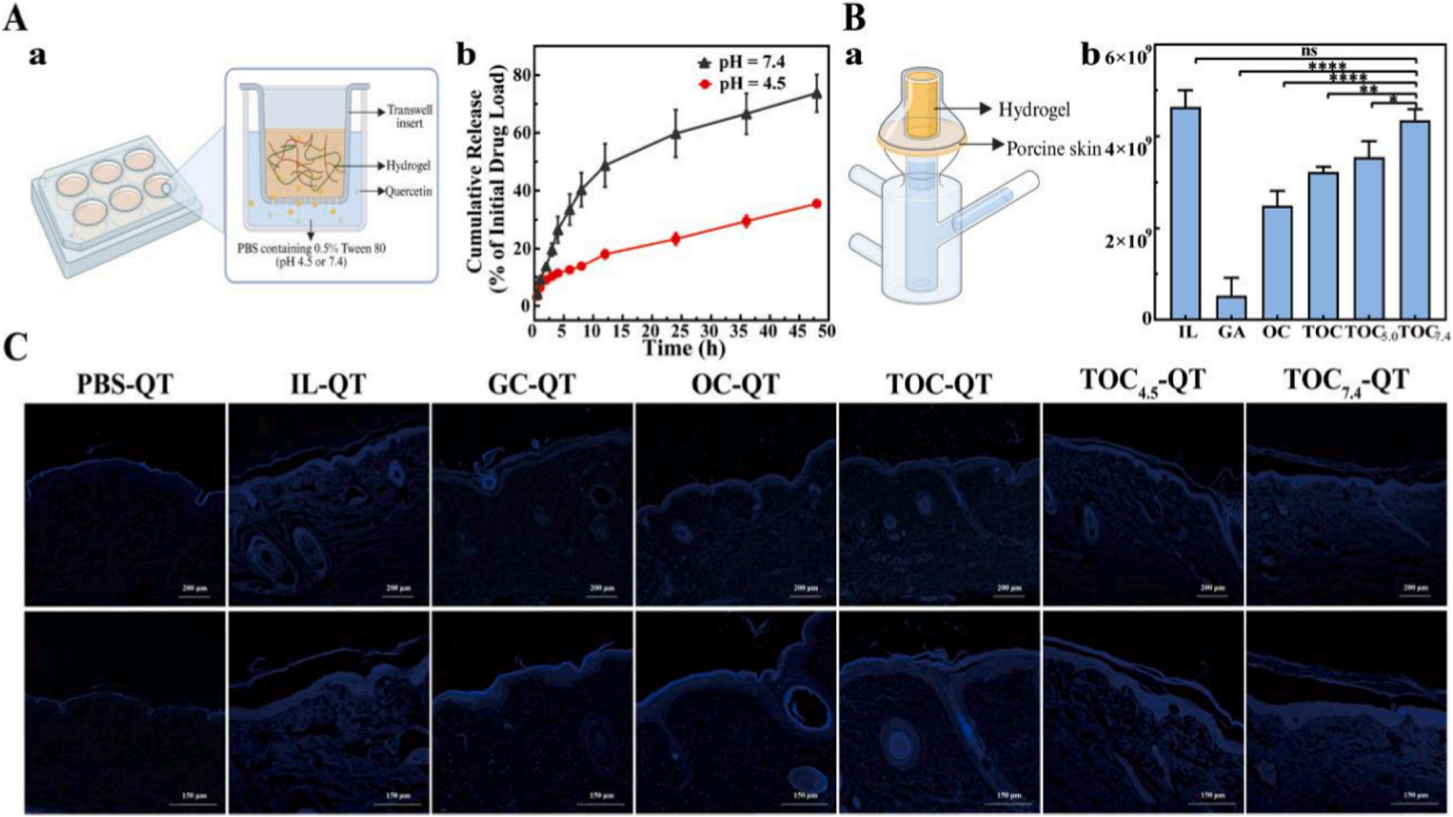
*In vitro* release and *ex vivo* skin permeation of TOC-QT hydrogels. (A) *In vitro* release behavior: (a) Transwell release setup, and (b) cumulative release profiles over 48 h. (B) *Ex vivo* skin permeation: (a) Franz diffusion cell setup, and (b) quantitative skin penetration intensity. (C) Representative transdermal fluorescence images after 24 h, where IL denotes the positive control group containing ionic liquid (scale bars: top = 200 μm, bottom = 150 μm). Data are presented as mean ± SD (n ≥ 3). Statistical significance was determined by one-way ANOVA followed by Dunnett's multiple comparisons test versus TOC (pH 7.4)(****p < 0.0001, ***p < 0.001, p < 0.01, *p < 0.05, and ns = not significant).

The TOC-QT hydrogel showed clear pH-responsive release behavior, with cumulative release at 24 h reaching 59.7% at pH 7.4 but only 23.3% at pH 4.5 (Fig. 4A). To gain insight into the release mechanism, the initial 12 h data were fitted with the Higuchi and Korsmeyer–Peppas models (Table 3 and Fig. S7). At pH 4.5, the Higuchi model showed a good fit (R^2^ = 0.968), and the Korsmeyer–Peppas exponent was 0.440 (R^2^ = 0.975), indicating diffusion-dominated release with Fickian characteristics. At pH 7.4, the Higuchi fit remained strong (R^2^ = 0.994), while the Korsmeyer–Peppas exponent increased to 0.607 (R^2^ = 0.991), suggesting a deviation from purely Fickian diffusion and a greater contribution from polymer relaxation. These results indicate that diffusion remains the dominant mechanism under both conditions, while polymer relaxation becomes more pronounced at pH 7.4. This trend is consistent with the pH-responsive swelling behavior of the hydrogel (Fig. 2), where deprotonation of carboxyl groups promotes network relaxation, increases water uptake, and reduces diffusional resistance, thereby facilitating quercetin release.

**Table 3:** Kinetic parameters of quercetin release from the hydrogels fitted by Higuchi and Korsmeyer-Peppas models (0–12 h).

Medium	Kinetic Model	Rate Constant (*k*)	Release Exponent (*n*)	R^2^
**pH 7.4**	Korsmeyer-Peppas	0.035	0.607	0.991
	Higuchi	-	-	0.994
**pH 4.5**	Korsmeyer-Peppas	0.056	0.440	0.975
	Higuchi	-	-	0.968

Ex vivo transdermal fluorescence imaging confirmed these trends (Fig. 4B and C). After 24 h, the PBS group showed minimal signal, whereas the ionic liquid (IL) control exhibited intense epidermal and dermal fluorescence. While GC-QT and OC-QT displayed weak or discontinuous deposition, TOC-QT achieved a highly uniform intradermal distribution, delivering a 6.2-fold increase in transdermal permeability compared to GC-QT. Specifically, TOC-QT at pH 7.4 yielded the strongest fluorescence among all hydrogels, outperforming the pH 4.5 group by 22.7% and closely approaching the positive IL control. These findings further support that TOC effectively enhances pH-responsive transdermal delivery, making it suitable for the near-neutral microenvironment of damaged skin.

### 2.7 Cytotoxicity

**Fig. 5:**
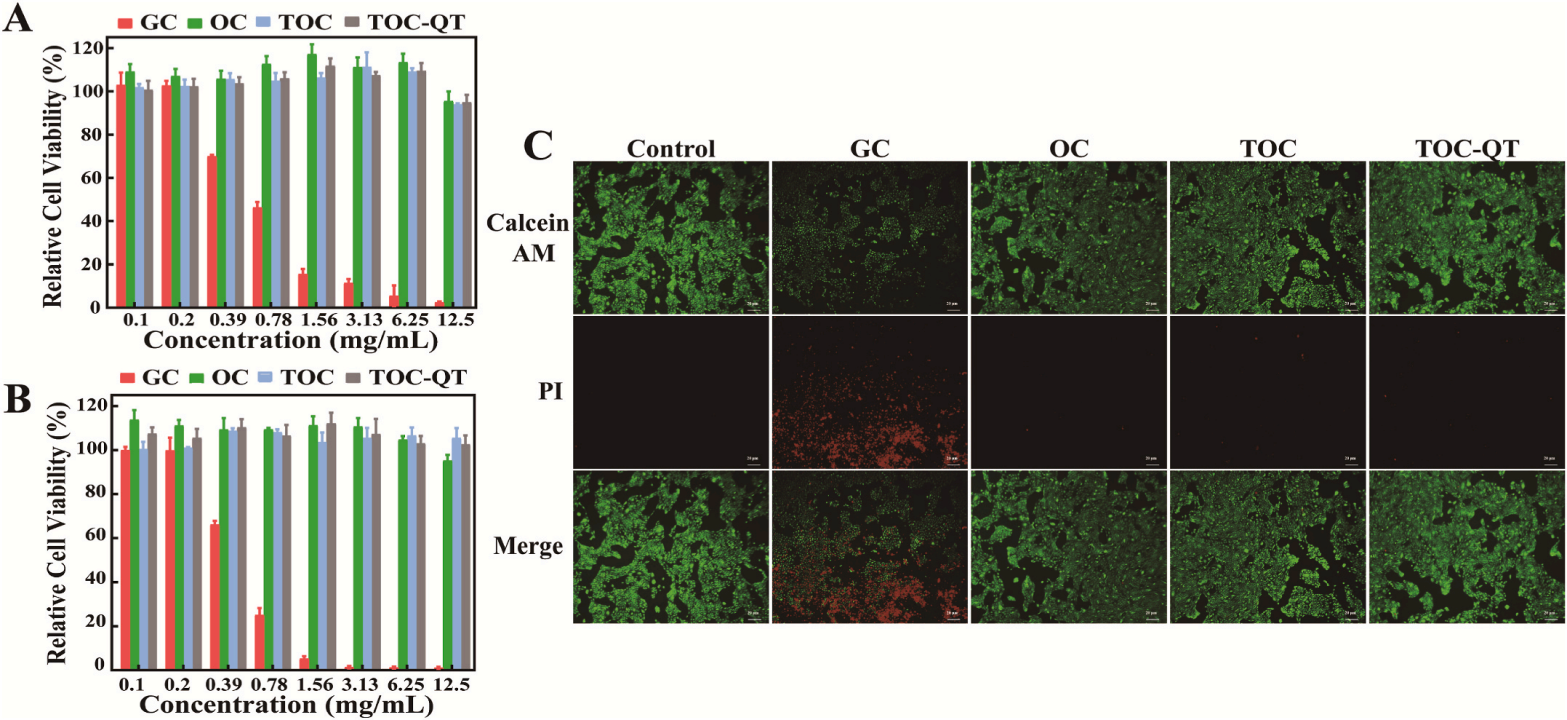
Cytocompatibility of hydrogels on HaCaT cells. (A) CCK-8 viability assay at 24 h and (B) 48 h. (C) Live/dead fluorescence imaging. Data represent mean ± SD (n = 3). Values are expressed as percentage of untreated control. Scale bar = 50 μm.

The CCK-8 assay and live/dead staining gave consistent results. GC showed cytotoxicity at low concentrations. Cell viability decreased significantly when the hydrogel concentration reached 0.39 mg/mL. In contrast, OC, TOC and TOC-QT were biocompatible. After 48 h of incubation with 12.5 mg/mL, cell viability remained close to the control value (Fig. 5A and B). Live/dead images further confirmed these findings (Fig. 5C).

### 2.8 Repair of UVB-damaged cells

**Fig. 6:**
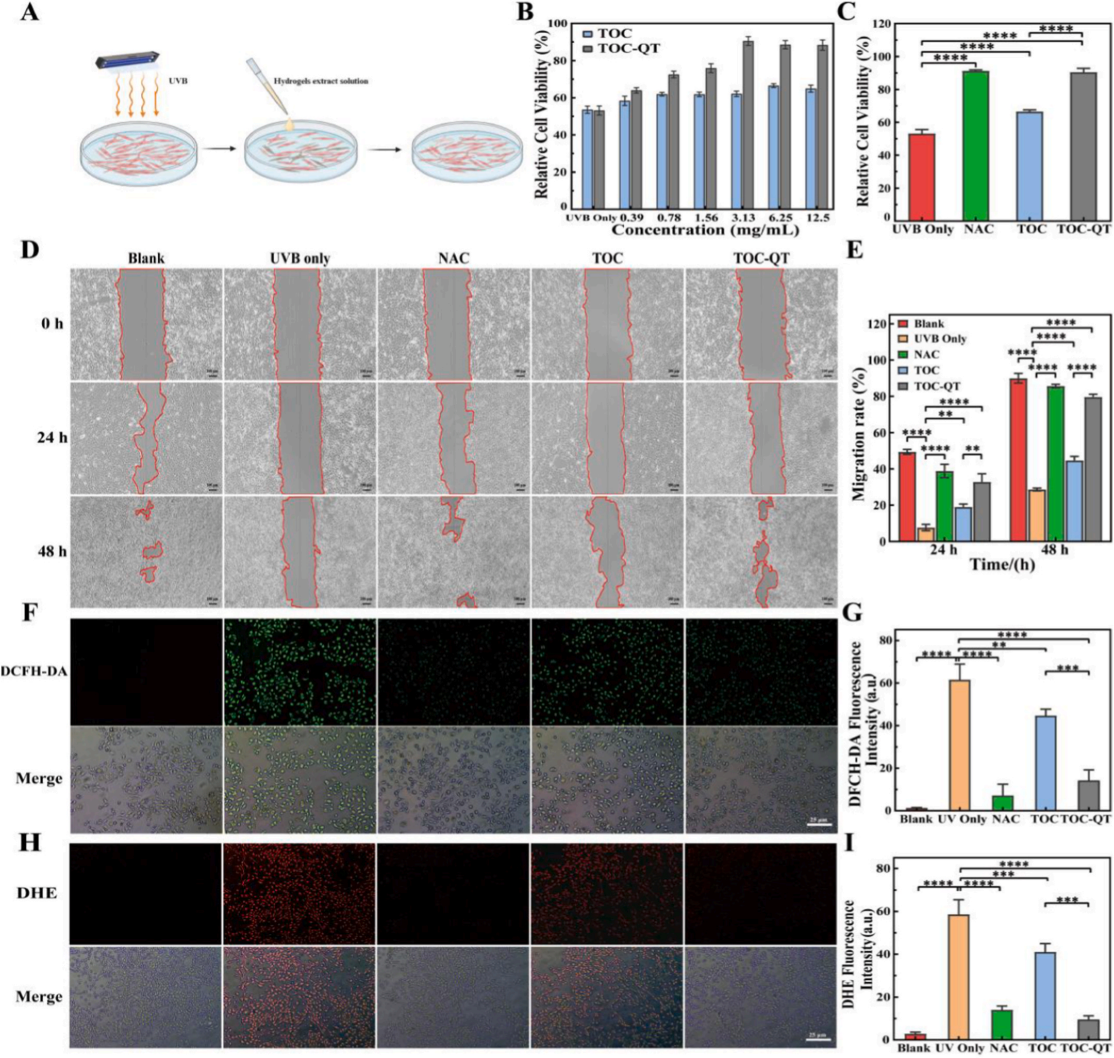
Hydrogel-mediated repair of UVB-induced damage in HaCaT cells. (A) Experimental design. (B) Dose-dependent effects of TOC and TOC-QT on HaCaT viability after UVB exposure. (C) Cell viability at selected concentrations (TOC: 6.25 mg/mL, TOC-QT: 3.13 mg/mL) and NAC (1 mM). (D) Representative scratch-wound images. (scale bar = 200 μm). (E) Quantification of wound closure. (F) Representative DCFH-DA images (scale bar = 25 μm). (G) DCFH-DA fluorescence quantification. (H) Representative DHE images (scale bar = 25 μm). (I) DHE fluorescence quantification. Data are presented as mean ± SD (n ≥ 3). ****p < 0.0001, ***p < 0.001, **p < 0.01, *p < 0.05.

A UVB damage model was established in HaCaT cells (Fig. 6A), with untreated healthy cells serving as the Blank group. N-acetylcysteine (NAC, 1 mM), a widely used antioxidant [59], served as the positive control (NAC group) to protect cells against UVB-induced oxidative stress. We tested the repair effect of TOC and TOC-QT at different concentrations. The CCK-8 assay showed that UVB strongly reduced cell viability. TOC at 6.25 mg/mL partially restored viability to about 66.6%. TOC-QT at 3.13 mg/mL restored viability to about 90.5%, similar to the NAC group (91.4%) (Fig. 6B and C).

In the scratch assay, UVB inhibited migration. After 48 h of treatment, wounds in the TOC-QT group were nearly closed. The wound area in TOC-QT was significantly smaller than in the UVB only group and smaller than in the TOC group (Fig. 6D and E). These data indicate that TOC-QT promotes migration and repair of damaged keratinocytes.

DCFH-DA and DHE staining showed increased intracellular ROS after UVB exposure. DCFH-DA readings were corrected for QT intrinsic fluorescence (Fig. S8). Using these corrected DCFH-DA values together with DHE results, TOC-QT reduced ROS accumulation, decreasing DCFH-DA and DHE fluorescence by 76.9% and 83.8%, respectively, compared with the UVB only group. These ROS levels were close to those of the NAC group. TOC also reduced ROS, but to a lesser extent than TOC-QT, with DCFH-DA and DHE fluorescence in the TOC group reduced by 68.2% and 77.2%, respectively, compared with the UVB only group (Fig. 6F–I). In summary, TOC-QT restored cell viability, enhanced migration, and reduced oxidative stress in UVB-injured HaCaT cells, supporting its reparative potential.

### 2.9 Protective effect against UVB-induced damage

**Fig. 7:**
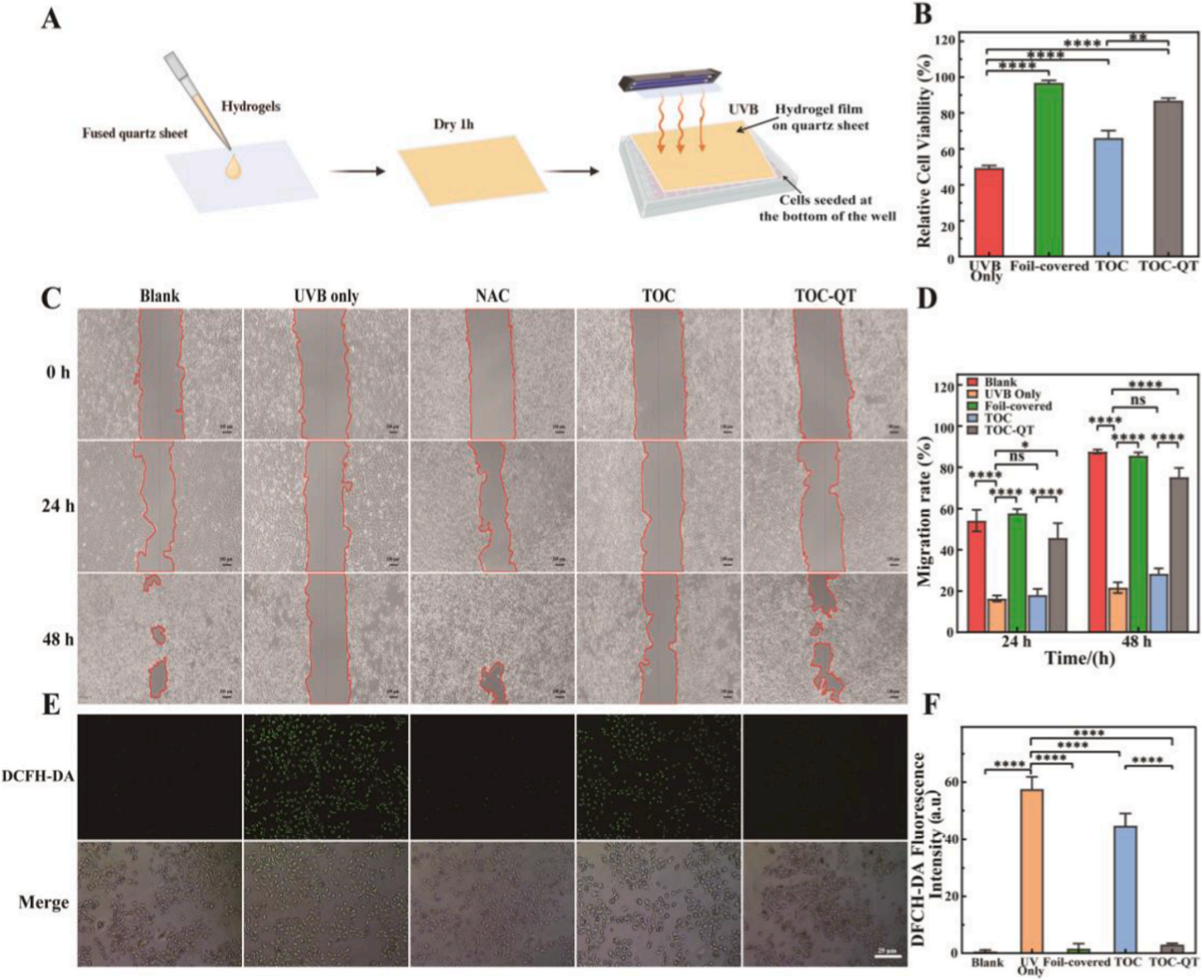
Hydrogel protection of HaCaT cells against UVB-induced damage. (A) Experimental design. (B) Cell viability after UVB exposure. (C) Representative scratch-wound images (scale bar = 200 μm). (D) Quantification of wound closure. (E) Representative DCFH-DA images (scale bar = 25 μm). (F) DCFH-DA fluorescence quantification. Data are presented as mean ± SD (n ≥ 3). ****p < 0.0001, ***p < 0.001, **p < 0.01, *p < 0.05.

QT provides strong UV absorption and imparts UV shielding capability [42,43] to the hydrogel. A 0.5 mm fused quartz sheet was used to support the hydrogel film, and cells were irradiated with UVB after film formation (Fig. 7A). Untreated healthy cells served as the Blank group, while cells exposed to UVB without any protection served as the UVB only group. Cells completely shielded with aluminum foil served as the Foil-covered group.

CCK-8 results showed that cell viability in the UVB only group remained low (49.4%) even with the quartz sheet barrier. The TOC film provided moderate protection, with viability increasing to 66.0%, likely due to the UV absorption of TFPs and CMCS together with the physical barrier of the hydrogel film. In contrast, the TOC-QT film showed stronger protection, and cell viability was comparable to that of the foil-covered group (Fig. 7B). Scratch assays and DCFH-DA staining showed similar trends. Under TOC-QT protection, cell migration after 48 h was largely restored and ROS production remained low (Fig. 7C–F). These results indicate that the TOC-QT film effectively shields cells from UVB-induced damage.

### 2.10 *In vivo* repair of UVB-induced skin damage

For biosafety assessment, blood compatibility and skin tolerance were evaluated. Hemolysis was <3% both before and after drug loading. Daily topical application for 7 days produced no visible skin lesions (Figs. S9 and S10).

**Fig. 8:**
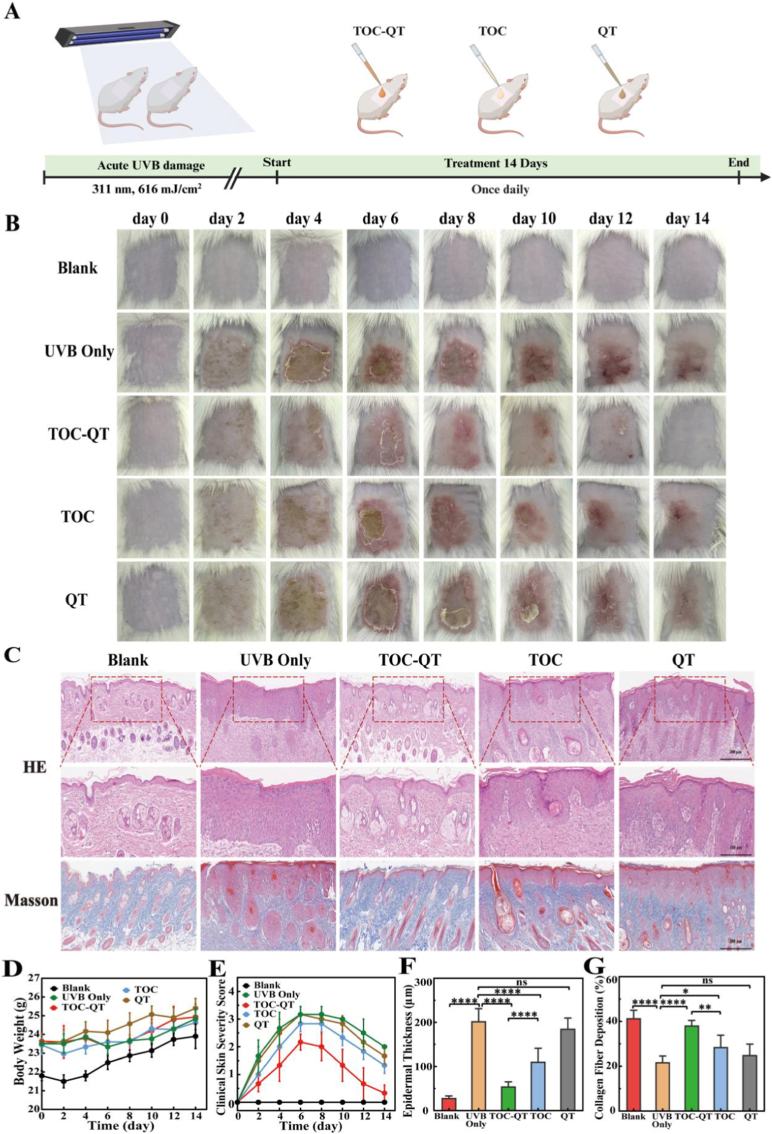
Therapeutic effects of hydrogels and quercetin solution on acute UVB-induced skin injury in mice. (A) Schematic illustration of the experimental design. (B) Representative dorsal skin images of the blank control, UVB, TOC hydrogel, TOC-QT hydrogel, and QT solution groups. (C) Representative H&E and Masson's trichrome staining images on Day 14. (D) Body weight changes during the treatment period. (E) Clinical lesion scores. (F) Epidermal thickness quantification. (G) Collagen-positive area quantification. Data are presented as mean ± SD. Body weight and clinical score: n ≥ 5 mice per group; histological quantification: n = 3 mice per group. ****p < 0.0001, ***p < 0.001, **p < 0.01, *p < 0.05.

Fig. 8A shows the experimental workflow. A BALB/c mouse model of UVB-induced skin injury was established on the dorsal skin. Mice were treated topically with blank TOC, TOC-QT (drug-loaded TOC), or a QT solution. After UVB exposure, the skin exhibited marked erythema, desquamation, and occasional ulceration (Fig. 8B). Body weight did not decrease significantly in any group during the study period (Fig. 8D).The QT solution produced minimal improvement, which may reflect its poor solubility. TOC provided moderate repair. By day 10, the clinical score of the TOC group was significantly lower than that of the UVB group, while TOC-QT produced the greatest improvement. TOC-QT enhanced quercetin solubility and transdermal delivery, reduced erythema and ulceration, and accelerated wound closure; wounds in the TOC-QT group were essentially closed by day 12 and skin appearance approached normal by day 14 (Fig. 8B and E).

H&E staining showed that UVB increased epidermal thickness to 202.5 μm and caused irregular elongation of the epidermal ridges. TOC-QT reduced epidermal thickness to 54.6 μm and restored basal layer morphology toward normal. TOC produced only partial recovery (epidermal thickness 110.9 μm). QT alone had limited effect (Fig. 8C and F). Masson's trichrome staining indicated that UVB decreased dermal collagen, with the collagen-positive area reduced to 21.8%. TOC-QT increased the collagen-positive area to 41.4%, a value significantly higher than that of the UVB group and not significantly different from the blank control; TOC and QT groups reached 28.5% and 24.9%, respectively (Fig. 8C and G). Overall, histological findings are consistent with the clinical phenotype and indicate that TOC-QT substantially promotes structural repair of UVB-damaged skin while maintaining biocompatibility.

### 2.11 Skin protection against UVB-induced damage

**Fig. 9:**
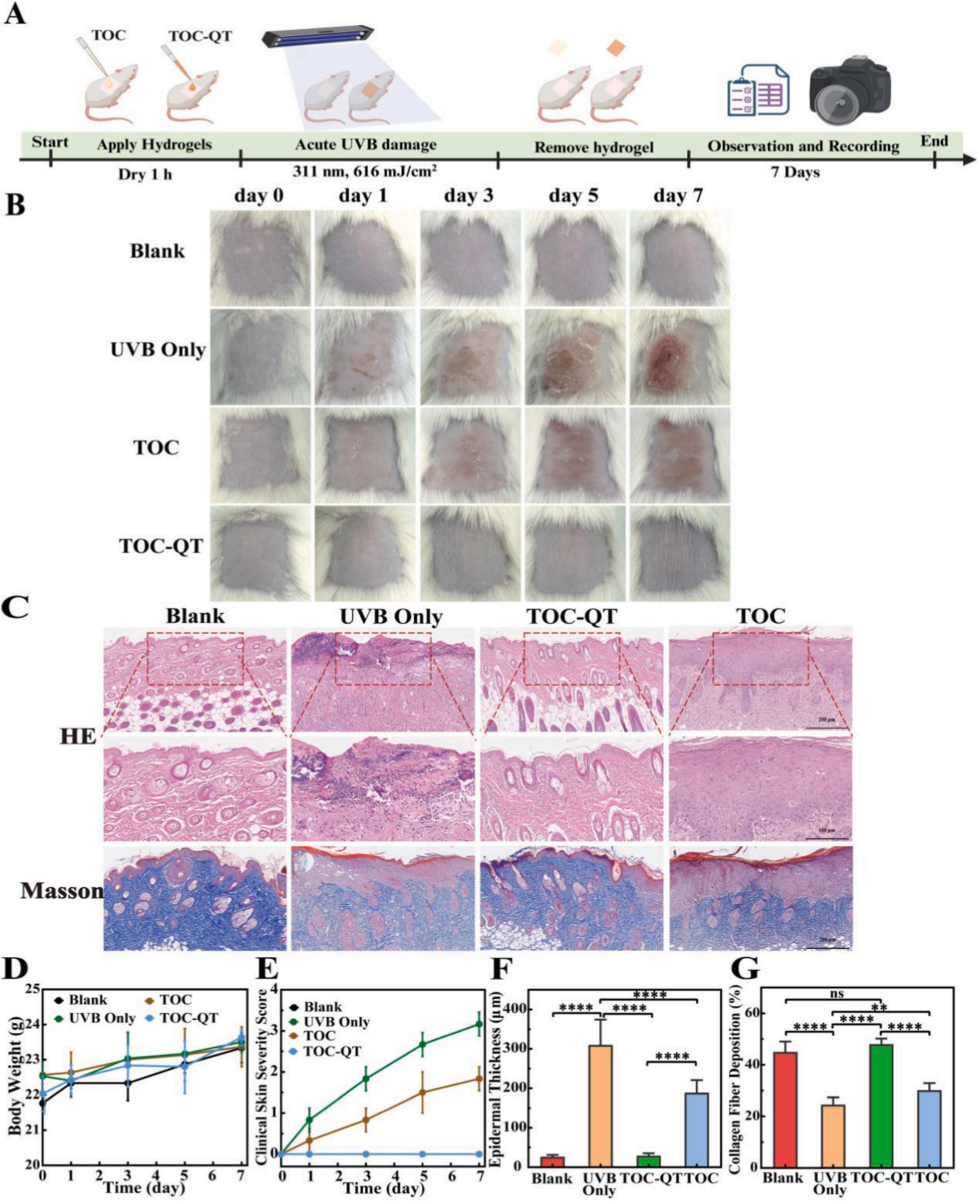
Preventive effect of hydrogels on acute UVB-induced skin injury in mice. (A) Experimental design. (B) Representative dorsal skin images. (C) Representative H&E and Masson's trichrome staining images on Day 7. (D) Body weight changes during the treatment period. (E) Clinical lesion scores. (F) Epidermal thickness quantification. (G) Collagen-positive area quantification. Data are presented as mean ± SD. Body weight and clinical score: n ≥ 5 mice per group; histological quantification: n = 3 mice per group. ****p < 0.0001, ***p < 0.001, **p < 0.01, *p < 0.05.

To assess skin protection, hydrogels were applied to dorsal skin before UVB exposure (Fig. 9A). Macroscopic observation and clinical scoring showed minimal damage in the TOC-QT group over the 7-day observation period. By contrast, the UVB group developed progressive desquamation and ulceration, while the TOC group showed only mild erythema without frank peeling (Fig. 9B and E).

H&E staining showed marked epidermal hyperplasia and dense cellular infiltration in the epidermis and superficial dermis in the UVB group, consistent with acute UVB-induced injury. In contrast, TOC-QT preserved a more intact epidermal structure and epidermal–dermal interface (Fig. 9C). Epidermal thickness was significantly reduced in the TOC group (188.8 μm) compared with the UVB group (309.5 μm), although still higher than the blank control (26.3 μm) (Fig. 9F). Morphologically, reduced cellular infiltration was observed in TOC-treated skin relative to UVB based on H&E staining. This observation is based on histological morphology and does not reflect specific inflammatory-cell subtypes. Masson's staining further showed improved collagen organization in TOC-QT, while UVB caused obvious collagen loss (Fig. 9C). Collagen-positive area was 30.1% in TOC versus 24.6% in UVB (Fig. 9G). No significant changes in body weight were observed among groups, suggesting no overt systemic toxicity (Fig. 9D).

These findings demonstrate that the TOC-QT hydrogel not only effectively repairs UVB-induced skin damage but also provides strong preventive protection when applied prior to UVB exposure.

## 3 Discussion

UVB irradiation disrupts the integrity of the stratum corneum, leading to increased transepidermal water loss, altered lipid organization, and enhanced inflammatory responses [5,7]. These changes disrupt the homeostasis of the skin acid mantle by impairing barrier integrity and altering the balance of acidic components, shifting the local microenvironment from its normal weakly acidic state toward near-neutral conditions. Similar pH shifts have also been reported in acute wounds and various dermatitis conditions associated with skin barrier impairment [8,9,60]. This barrier-associated near-neutral microenvironment provides a physiological basis for developing pH-responsive biomaterials for photodamaged skin. Recent studies suggest that biomaterials capable of responding to pathological microenvironments, rather than serving only as passive dressings, represent an important direction for the development of precision therapies [14].

The TOC hydrogel exhibited distinct pH responsiveness under near-neutral conditions, indicating that its swelling behavior was regulated not by a single ionizable group but by the collective effects of the polysaccharide network microenvironment [61]. Although the theoretical pKa of glucuronic acid residues is approximately 3.5 [62], the local charge distribution, physical chain entanglement, and ion-screening effects within the polyelectrolyte network can shift the apparent dissociation constant (pK_a_^app^) toward a higher pH [61,63]. As the pH increased, amino group deprotonation and enhanced carboxyl group dissociation in CMCS increased the net negative charge of the network. This promoted water uptake and pore expansion through electrostatic repulsion between polymer chains and Donnan osmotic pressure [61,64]. The swelling of the TOC hydrogel was markedly reduced after the addition of 0.9% NaCl (Fig. 2B), confirming that external ions weakened these driving forces by shielding the network charges [65,66]. During hydration, the dynamic rearrangement of the hydrogen-bonding network reduced local chain constraints and improved network adaptability, further promoting the swelling response [67,68].

Notably, the TOC hydrogel maintained an intact gel structure even under highly swollen conditions, demonstrating excellent network stability. This behavior is attributed to the hierarchical framework formed by dynamic Schiff base bonds, hydrogen bonding, and the physical chain entanglement of native high-molecular-weight TFPs [69]. Compared with the OC network composed only of oxidatively degraded short chains, the introduction of native TFPs enhanced physical chain entanglement and facilitated effective stress transfer [70,71], thereby compensating for the loss of network continuity caused by oxidation and improving the overall toughness of the hydrogel. Although the high density of aldehyde groups introduced by oxidation endowed the material with enhanced environmental responsiveness, the pronounced molecular weight reduction (from 1079.6 to 5.0 kDa) could compromise network integrity.

This hierarchical network architecture was further supported by microstructural observations. The continuous and highly interconnected porous structure helped distribute local stress during swelling, while the wrinkled pore walls provided additional space for deformation, reducing structural instability caused by rapid water uptake [72,73] (Fig. 1E and F). Rather than simply increasing the crosslinking density, the physical chain entanglement retained in the semi-IPN allowed polymer chains to slide and rearrange under external stress, dissipating mechanical energy through reversible chain motion and thereby reducing the tendency for brittle fracture [35,74]. This mechanism is consistent with the wider linear viscoelastic region and higher compressive deformability observed for the TOC hydrogel (Fig. 1C and D). Combined with the swelling results, these findings further demonstrate that the TOC hydrogel maintained good structural integrity even under highly hydrated conditions (Fig. 2A). The introduction of native TFPs therefore achieved a balance between environmental responsiveness and structural stability. The dynamic crosslinking provided by OTFPs acted synergistically with the toughness and water retention imparted by native TFPs. This structural advantage provides a foundation for subsequent drug loading, controlled release, and transdermal delivery.

The high water retention capacity of the TOC hydrogel helped fully hydrate the stratum corneum and reduce the diffusion barrier, thereby promoting drug penetration [75,76]. CMCS formed hydrogen bonds and other weak intermolecular interactions with the polyphenolic structure of QT, reducing molecular aggregation and improving its apparent solubility [77,78]. In addition, CMCS provided the hydrogel matrix with a certain degree of UV-shielding capability [29]. Under near-neutral conditions, the TOC semi-IPN underwent controlled swelling, providing dynamically adjustable diffusion pathways for QT. Release kinetic analysis showed that QT release followed a Fickian diffusion mechanism at pH 4.5 but shifted to an anomalous transport mechanism governed by both diffusion and polymer chain relaxation at pH 7.4 (Table 3). Enhanced network relaxation and pore expansion under near-neutral conditions reduced mass transfer resistance, thereby promoting QT release and improving transdermal delivery efficiency [79]. The near-neutral conditions associated with skin barrier impairment were sufficient to trigger a pronounced swelling response in the TOC hydrogel. Compared with pH 4.5, the maximum swelling ratio and the 24 h cumulative QT release at pH 7.4 increased by approximately 1.5-fold and 2.6-fold, respectively. These results indicate that the barrier-associated near-neutral microenvironment can induce substantial network restructuring and effectively enhance drug transport.

The therapeutic efficacy of the TOC-QT hydrogel in the UVB-induced photodamage model further demonstrated that the advantages of drug solubilization, controlled release, and transdermal delivery could be translated into clear biological benefits. At the cellular level, the uniform dispersion and improved apparent solubility of QT within the hydrogel enhanced its accessibility to cells, thereby mitigating UVB-induced oxidative stress, as evidenced by reduced ROS accumulation, higher cell viability, and accelerated cell migration. In vivo, these advantages, together with the controlled release and transdermal delivery capacity of the TOC hydrogel, promoted local drug accumulation, thereby accelerating inflammation resolution and improving the organized reconstruction of collagen fibers.

Although the design of the semi-IPN hydrogel was based on the physiological pH characteristics of human skin, the skin surface pH of mice was not monitored in situ during the experiments. This limitation is common in rodent studies, as hair removal can disrupt the stratum corneum and interfere with baseline skin surface pH measurements [[80], [81], [82]]. Furthermore, the skin surface pH and acidification mechanisms of mice differ from those of humans, making it difficult to directly relate the local pH changes after UVB irradiation to the human skin microenvironment [3,83,84]. Future research should combine precise real-time skin surface pH monitoring with hydrogel evaluation to further clarify the relationship among network response, drug transport, and the pathological microenvironment. In conclusion, the homologous TFPs/OTFPs semi-IPN hydrogel integrates near-neutral pH responsiveness with network stabilization, providing a localized therapeutic platform with improved structural stability, drug delivery capability, and photoprotective function.

## 4 Conclusion

In this work, we developed an all-natural semi-IPN hydrogel by integrating native TFPs and oxidized TFPs with CMCS. This design compensates for the structural limitations associated with polysaccharide oxidation while retaining the reactive functionality of OTFPs, resulting in improved gelation, flexibility (96.6% strain), and water retention. The hydrogel exhibited well-defined near-neutral pH responsiveness, showing accelerated swelling and controlled QT release under conditions relevant to damaged skin. CMCS enabled efficient loading of poorly soluble QT, and the hydration-rich network achieved a 6.2-fold increase in transdermal delivery. Biological studies demonstrated that the QT-loaded hydrogel enhanced keratinocyte migration, reduced oxidative stress by 76.9%, and protected cells from UVB-induced damage. In a murine model, the hydrogel accelerated structural skin repair and provided effective photoprotection. Taken together, this all-natural semi-IPN platform integrates near-neutral pH responsiveness, enhanced hydration, and efficient drug delivery, providing a promising strategy for UV-induced skin protection and repair.

## 5 Materials and methods

### 5.1 Materials

The *Tremella fuciformis* polysaccharides (TFPs; CAS No. 9075-53-0, purity ≥ 90%) used in this study were procured from Shanghai Macklin Biochemical Co., Ltd. Hydrogen peroxide (H_2_O_2_, 30% w/w), hydroxylamine hydrochloride, copper(II) sulfate pentahydrate (CuSO4·5H_2_O), glyoxal (40% w/w) and Quercetin (purity ≥ 98%) were obtained from Aladdin Biochemical Technology Co., Ltd. Carboxymethyl chitosan (CMCS; substitution degree ≥ 80%, Mw: 100–200 kDa) and glutaraldehyde were obtained from Shanghai Macklin Biochemical Co., Ltd. The human epidermal keratinocyte cell line (HaCaT) was sourced from Procell Life Science & Technology Co., Ltd. Dulbecco's Modified Eagle Medium (DMEM), RPMI-1640, fetal bovine serum (FBS), phosphate-buffered saline (PBS), and penicillin-streptomycin solution were purchased from Gibco (Thermo Fisher Scientific). Dialysis bags (3.5 kDa molecular-weight cutoff) were acquired from Shanghai Yuanye Bio-Technology Co., Ltd. The Cell Counting Kit-8 (CCK-8) assay kit, The reactive oxygen species (ROS) assay kit and Live/Dead cell staining kit were supplied by Xiamen Haibio Biotechnology Co., Ltd. All chemicals were of analytical grade, and deionized water was used throughout the experiments.

### 5.2 Preparation of oxidized Tremella polysaccharides (OTFPs)

TFPs were oxidized with 6.7% (w/v) H_2_O_2_ and 0.01% (w/v) CuSO_4_ at 40 °C under agitation for 12 h. The reaction mixture was dialyzed in ultrapure water using a dialysis bag (MWCO 3500 Da) for 72 h, with water changes three times per day, and then freeze-dried to yield OTFPs. Carboxyl content was determined by the calcium acetate titration method, and aldehyde content was quantified using a UV–Vis-based hydroxylamine method as described in the Supporting Information (Section S1.1). Molecular weight data were adopted from our previously reported characterization of the same polysaccharide batch [31], which used identical oxidation conditions and gel permeation chromatography parameters.

### 5.3 Hydrogel preparation

All hydrogel formulations were prepared at room temperature (26 ± 1 °C) using cylindrical molds (diameter: 1.5 cm, height: 0.5 cm) to ensure consistent sample geometry. Stock solutions of TFPs, OTFPs, CMCS, and glyoxal (GA) were separately prepared in ultrapure water under magnetic stirring until complete dissolution. Detailed formulations, final component concentrations, and gelation times are summarized in Table 2.

For Glyoxal/CMCS (GC) hydrogel preparation, the CMCS solution was mixed with the GA solution under gentle stirring and allowed to stand at room temperature until complete gelation.

For OTFPs/CMCS (OC) hydrogel preparation, the OTFPs solution was combined with the CMCS solution and maintained at room temperature until formation of a single-network hydrogel.

For TFPs/OTFPs/CMCS (TOC) hydrogel preparation, TFPs/OTFPs blends at predetermined ratios were first prepared and subsequently mixed with CMCS under gentle stirring to obtain a semi-interpenetrating network (semi-IPN) hydrogel.

For drug-loaded hydrogel (TOC-QT) preparation, QT was first dispersed in the CMCS solution and then mixed with the TFPs/OTFPs blend solution. The resulting mixture was maintained at room temperature to achieve complete gelation.

Unless otherwise specified, all precursor solutions were freshly mixed immediately prior to gelation using identical total volumes and the molds described above. Gelation time was determined using the vial inversion method.

### 5.4 Quantification of quercetin solubility and hydrogel loading

UV–Vis spectrophotometry at 377 nm, together with a standard calibration curve (Fig. S1), was used for the quantification of QT concentration. For solubility measurements, excess QT was dispersed in CMCS solutions (5% and 10%, w/v), followed by magnetic stirring and centrifugation (10,000 rpm, 10 min) to remove undissolved solids. Prior to analysis, the supernatant was diluted with a pH 7.4 PBS solution containing 0.5% (v/v) Tween 80 to ensure compatibility with the established analytical system. The QT concentration was determined using a calibration curve constructed in the corresponding pH 7.4 PBS containing 0.5% Tween 80 medium. Detailed method validation is provided in the Supporting Information (Table S1).

For the drug-loaded hydrogel (TOC-QT), the QT content was evaluated by measuring its concentration in the CMCS precursor solution using the same analytical procedure, including the identical dilution and measurement conditions. The hydrogel was prepared via in situ gelation under closed conditions without washing or purification steps, and no visible precipitation or phase separation was observed during or after gel formation. Therefore, the drug loading within the hydrogel matrix was estimated based on the experimentally determined QT concentration in the precursor solution and the predefined formulation ratio. All formulations and component concentrations are summarized in Table 2.

### 5.5 Characterization of hydrogels

#### 5.5.1 Fourier transform infrared (FT-IR) spectroscopy

Prior to FT-IR analysis, all hydrogel samples were completely freeze-dried and ground into fine powders to ensure homogeneous contact during measurement. The spectra were recorded using a Nicolet 6700 Fourier transform infrared spectrometer in attenuated total reflection (ATR) mode over the wavenumber range of 4000–500 cm^−1^at room temperature. Each spectrum was collected to evaluate the characteristic functional groups and interactions within the hydrogel network.

#### 5.5.2 Morphological observation

To visualize the internal porous morphology of the hydrogels, the freshly prepared samples were first frozen at −80 °C and subsequently lyophilized for 48 h to remove water while maintaining the porous architecture as much as possible. The freeze-dried hydrogels were carefully fractured in liquid nitrogen to expose the internal cross-sections. The fractured samples were mounted onto aluminum stubs using conductive carbon tape and sputter-coated with a thin layer of gold prior to imaging. The internal microstructure of the hydrogels was observed using a Hitachi Regulus 8100 scanning electron microscope operated at an accelerating voltage of 3 kV.

To quantitatively evaluate the microstructural parameters, the obtained SEM images were analyzed using ImageJ software (National Institutes of Health, USA). For each sample, the average pore size was determined by measuring at least 50 random pores, and the pore-wall thickness was calculated based on 12 random measurements from multiple representative SEM micrographs. The porosity of the hydrogel scaffolds was quantified by converting the cross-sectional SEM images into binary images via threshold adjustment in ImageJ, and calculating the area fraction of the porous voids relative to the total statistical area.

#### 5.5.3 X-ray diffraction (XRD)

Freeze-dried powders were analyzed on a Bruker D8 Advance X-ray diffractometer over 2θ = 5–60°.

#### 5.5.4 Water evaporation tests and contact angle measurements

The water evaporation rates and water contact angles of various hydrogels were determined according to the detailed procedures described in the Supporting Information (Sections S1.3 and S1.4).

#### 5.5.5 TGA

TGA of pure QT powder and the hydrogels was performed on a Q500 analyzer (TA Instruments, USA).The lyophilized hydrogel samples and QT powder were directly placed into the sample pan. The analysis was conducted under a nitrogen atmosphere. Before the heating run, the system was purged with nitrogen at a flow rate of 50 mL/min for 5 min to ensure a stable inert environment. The samples were then heated from 30 °C to 600 °C at a constant heating rate of 20 °C/min to obtain the TG and DTG profiles.

#### 5.5.6 X-ray Photoelectron spectroscopy (XPS)

XPS measurements were performed using a Thermo Scientific ESCALAB 250 spectrometer equipped with a monochromatic Al Kα X-ray source. Survey and high-resolution spectra were collected in constant analyzer energy mode at pass energies of 150 and 30 eV, respectively. Binding energies were calibrated using the C 1s peak at 284.8 eV. Peak deconvolution was performed using Avantage software.

### 5.6 Mechanical testing

#### 5.6.1 Compressive testing

Compressive performance was measured using a universal testing machine (CMT6103, China Metes Industrial Systems, China). Prepare cylindrical samples (d = 20 mm, h = 5 mm). Compress at 1.0 mm/min and record the stress–strain curve.

#### 5.6.2 Rheological properties

Hydrogel rheological properties were evaluated using a rheometer (Haake Mars40, Thermo Fisher Scientific, Karlsruhe, Germany). Mold hydrogels into disk samples (d = 2 cm, h = 1 mm). Perform oscillatory strain sweep tests at 25 °C and 10 Hz, with a strain range of 1–1000%, to determine storage modulus (G’) and loss modulus (G”).

### 5.7 pH-responsive properties

#### 5.7.1 Swelling test

Freeze-dried hydrogels (d = 12 mm) were weighed (Wd) and incubated in 5 mL PBS (pH 4.5 or 7.4) with or without 0.9% NaCl at 37 °C. Samples were removed at predetermined intervals up to 48 h for pure PBS and 72 h for PBS with 0.9% NaCl, blotted lightly and weighed (Wt).Swelling ratio (SR, %) was calculated as:

(1) SR(%)=(Wt‐Wd)/Wd×100%

The pH-responsive index (PRI) was calculated using the maximum swelling values:

(2) $$\text{PRI}=\left({\text{SR}}_{7.4\;\left(\max\right)}\;‐\;{\text{SR}}_{4.5\;\left(\max\right)}\;\right)\;/\;{\text{SR}}_{5.0\;\left(\max\right)}$$

#### 5.7.2 *In vitro* drug release

For release testing, 200 μL hydrogel was placed in Transwell inserts and immersed in 1.2 mL receptor medium (PBS at pH 4.5 or 7.4, containing 0.5% Tween 80) at 37 °C [85]. At predetermined time intervals (0.5–48 h), aliquots were withdrawn and replaced with equal volumes of fresh medium to maintain sink conditions. Quercetin concentration was quantified by UV–Vis spectrophotometry, and the analytical method together with validation details is provided in the Supporting Information (SI). Cumulative release (%) was calculated based on the initial drug content and reported as mean ± SD (n = 3).

To gain insight into the release mechanism, kinetic modeling was performed using the initial 12 h release data, which correspond to the linear diffusion-dominated stage before equilibrium effects become significant. The data were fitted using the Higuchi and Korsmeyer–Peppas models:

(3) $$M_{t}/M_{\infty }=\text{kH}\;t^{1/2}$$

(4) $$M_{t}/M_{\infty }=k\;t^{n}$$

where M_t_/M_∞_ represents the cumulative release fraction at time t, kH and k are the release constants, and n is the release exponent reflecting the release mechanism. All fittings were performed using Origin software.

#### 5.7.3 *Ex vivo* transdermal penetration

Full-thickness porcine skin was obtained from the dorsal region of Bama miniature pigs (Taizhou Taihe Biotechnology Co., Ltd., Taizhou, China) and used to evaluate *ex vivo* transdermal penetration. Prior to the experiment, the skin samples were pre-equilibrated in PBS at the target pH (4.5 or 7.4) for 3 h to stabilize the skin surface microenvironment. Hydrogels with different quercetin loads were then uniformly applied to the epidermal side and allowed to gel in situ before mounting onto Franz-type diffusion cells (TP-12, Tianjin Jingtuo Instrument Co., Ltd., Tianjin, China). The receptor chamber was filled with PBS at the corresponding pH, and the entire system was maintained at 37 °C with continuous stirring at 350 rpm. During the 24 h incubation, a small volume of PBS at the matching pH was periodically added to the donor side to maintain hydrogel hydration and help maintain the local pH environment on the skin surface without disturbing the hydrogel structure. After 24 h, the skin samples were harvested and subjected to cryosectioning. The transdermal distribution of QT was visualized by confocal laser scanning microscopy (CLSM, FV3000, Olympus, Japan), and the obtained images were quantitatively analyzed using Fiji software. Fluorescence quantification followed a uniform image-processing workflow. Images were acquired with identical exposure settings. For each cryosection, a region of interest (ROI) of equal thickness covering the epidermis and upper dermis was selected. Background signal was subtracted and the total fluorescence intensity within the ROI was calculated.

### 5.8 *In vitro* UV absorption

UV–Vis spectra of hydrogels with or without quercetin were recorded using a Thermo Scientific microplate reader (Multiskan SkyHigh, Thermo Fisher Scientific, Waltham, MA, USA).

### 5.9 *In vitro* hydrogel degradation

The *in vitro* degradation behavior was evaluated according to a previously reported method with minor modifications [86,87]. Briefly, the cylindrical hydrogels (diameter: 1.5 cm, height: 0.5 cm) were incubated in pure PBS (pH 7.4) or PBS containing 0.5 mg/mL lysozyme at 37 °C for up to 7 days. At each predetermined time point, the hydrogels were collected and thoroughly washed with deionized water to remove residual buffer salts. Subsequently, the samples were lyophilized to measure their average dry weights. The degradation kinetics were expressed as the remaining weight percentage (%) over time (d), which was determined relative to the initial dry mass of the hydrogels at day 0.

### 5.10 Cell experiments

Cell culture procedures are described in the Supporting Information.

#### 5.10.1 Cytotoxicity

To evaluate the cytocompatibility of soluble components and potential degradation-related species, freeze-dried hydrogels were ground into a fine powder and extracted in the cell culture medium to obtain a precise concentration gradient (0.1–12.5 mg/mL). The resulting extracts were filtered to remove insoluble residues before cell exposure. HaCaT cells (8000 cells/well) were seeded in 96-well plates and incubated for 24 h, followed by treatment with the hydrogel extracts for another 24 h. Subsequently, 10% CCK-8 reagent was added to each well, and the absorbance at 450 nm was measured using a microplate reader (Multiskan GO 1510, Thermo Fisher Scientific, Waltham, MA, USA). Calculate relative cell activity (%) using the following formula:

(5) $$\text{Relative}\;\text{cell}\;\text{activity}\;\left(\%\right)=\left(A_{1}\;‐\;A_{0}\right)\;/\;\left(A_{2}\;‐\;A_{0}\right)\times 100\%$$

Where A_1_ and A_2_ are the absorbance of the treated and control cells, A_0_ is the absorbance of the blank well.

#### 5.10.2 Live/dead staining

Cells were seeded in 24-well plates, treated with hydrogel solutions for 24 h, and stained according to the manufacturer's instructions. Images were acquired using an inverted fluorescence microscope (Feica DM IL) equipped with a DFC450C digital camera (Leica Microsystems, Wetzlar, Germany).

#### 5.10.3 Photoprotection and repair assays

The UVB-induced damage model followed our previous report [31], with NAC as a positive control. For the protection model, hydrogel films (1.7 mm thick) were formed on quartz plates. After UVB irradiation, cells were incubated with different treatments for 24 h, and viability was assessed by CCK-8.

#### 5.10.4 Scratch assay

HaCaT cells (2.4 × 10^5^ cells/well) were seeded in 6-well plates. At 80–90% confluence, linear scratches were created using a 200 μL pipette tip. Cells were cultured in DMEM containing 2% FBS with or without treatments. After UV irradiation, treatments were added with a final volume of 2.0 mL per well. Images were captured at 0, 12, and 24 h using a Leica DMil inverted phase-contrast microscope under identical settings. Wound closure was quantified by Fiji based on the initial scratch area at 0 h.

(6) $$\text{Wound}\;\text{closure}\;\%=\left({\text{Area}}_{0}\;‐\;{\text{Area}}_{t}\right)\;/\;{\text{Area}}_{0}\times 100\%$$

#### 5.10.5 ROS assay

Dichlorodihydrofluorescein diacetate (DCFH-DA) and dihydroethidium (DHE) were used for ROS detection. Cells were irradiated with UVB, treated for 24 h, and stained according to the manufacturer's instructions. Fluorescence images were acquired using an inverted fluorescence microscope (Feica DM IL) equipped with a DFC450C digital camera (Leica Microsystems, Wetzlar, Germany).

### 5.11 Animal experiments

#### 5.11.1 Animals

Male BALB/c mice (SPF grade, 6–7 weeks old) were purchased from Guangdong Yaokang Biotechnology Co., Ltd. (license No. SCXK (Yue) 2025-0054). The animals were acclimated for 7 days before experiments.All animal procedures were performed in accordance with the national guidelines for the care and use of laboratory animals and were approved by the local Institutional Animal Care and Use Committee (IACUC) (approval No. 2025435-1).

#### 5.11.2 UVB model

One day before the experiment, the dorsal hair of mice was shaved to expose a 1.5 × 1.0 cm area. On day 0, mice (except for the blank group) were anesthetized and exposed to UVB irradiation at 0.57 mW/cm^2^ for 18 min (total dose ≈ 0.51 J/cm^2^). Animals were randomly assigned to different groups (n = 5 per group).

#### 5.11.3 Treatment regimen

Protection model: TOC or TOC-QT hydrogels were topically applied at a controlled volume of 200 μL over the exposed dorsal area (1.5 × 1.0 cm), forming a uniform layer with an approximate thickness of 1 mm. The hydrogel was applied 30 min before UVB exposure and allowed to gel in situ; residual hydrogel was gently removed after irradiation.

Repair model: UVB irradiation was performed first, followed by daily topical application of TOC, TOC-QT, or QT hydrogels. Dorsal skin was photographed at designated time points.

Clinical skin severity was evaluated in a blinded manner using a 0–4 scoring system based on visible erythema, scaling, crusting, and wrinkling. Detailed scoring criteria are provided in the Supporting Information (Section S1.10).

#### 5.11.4 Histological analysis

At predetermined endpoints (Days 7 and 14), mice were euthanized and full-thickness skin samples (1.0 × 1.0 cm) were collected from the treated area. Three representative animals from each group were randomly selected for histological and quantitative analysis. Samples were processed for hematoxylin and eosin (HE) and Masson's trichrome staining. Histological quantification was performed using ImageJ software. Epidermal thickness was determined by measuring the vertical distance from the basal layer to the stratum corneum across multiple random fields. The collagen-positive area (%) was quantified from Masson's trichrome images by calculating the percentage of the blue-stained collagen area relative to the total dermal area.

### 5.12 Statistical analysis

Data are presented as mean ± SD of independent biological replicates (n ≥ 3, as indicated in each figure). Statistical analyses were performed using GraphPad Prism 9 (GraphPad Software, San Diego, CA, USA). For comparisons among multiple groups, one-way analysis of variance (ANOVA) was performed, followed by Tukey's post hoc test for all pairwise comparisons or Dunnett's multiple comparisons test for comparisons against the control group. For comparisons between two independent groups, an unpaired two-tailed Student's t-test was applied. All statistical tests were two-tailed. A p-value < 0.05 was considered statistically significant.

## CRediT authorship contribution statement

**Fei Lin:** Conceptualization, Data curation, Formal analysis, Validation, Writing – original draft. **Huimin Xie:** Formal analysis, Software. **Fang Luo:** Formal analysis. **Zhenyu Lin:** Validation. **Yong Wang:** Validation. **Bin Qiu:** Conceptualization, Project administration, Supervision, Writing – review & editing. **Yanjing Huang:** Resources, Supervision.

## Declaration of competing interest

The authors declare the following financial interests/personal relationships which may be considered as potential competing interests: Bin Qiu reports financial support was provided by Scientific and Technological Foundation of Fujian Province of China. Bin Qiu reports financial support was provided by Science and Technology, Fujian Province. If there are other authors, they declare that they have no known competing financial interests or personal relationships that could have appeared to influence the work reported in this paper.

## Data Availability

Data will be made available on request.

## Supplementary data

Supplementary data to this article have been uploaded.
