# Supplementary material for: Homologous Tremella fuciformis semi-IPN hydrogels with entanglement-assisted network stabilization for near-neutral pH-responsive photodamage therapy
Source: Mater Today Bio. 2026 Aug 4;40:103503. doi: 10.1016/j.mtbio.2026.103503 (PMC13475242; doi:10.1016/j.mtbio.2026.103503)
Supplement: Supplementary file 1 [file mmc1.doc]

**Supporting information**

**S1. Methods**

**S1.1 Characterization of Oxidized Tremella Polysaccharides (OTFPs)**

**S1.1.1 Determination of carboxyl content**

The carboxyl content of OTFPs in this study was quantified using the calcium acetate method. The polysaccharide samples were reacted with a calcium acetate solution, which releases acetic acid. Phenolphthalein was utilized as a color indicator, and the mixture was titrated using a standard sodium hydroxide solution. Native TFPs were employed as a blank control group. The total carboxyl mass percentage was calculated according to the following equation:

COOH (%) = [ ( *V*_NaOH_ - *V*_0_ ) × 0.01 × 45 ] *m* / × 100%	(1)

where 0.01 is the concentration of NaOH (mol/L), *V*_NaOH_ is the volume of NaOH solution (mL), *V*_0_ is the control (mL), *m* is the sample weight (g), and 45 is the molecular weight of the carboxyl group.

**S1.1.2 Determination of aldehyde content**

The aldehyde content was quantified by reacting the samples with hydroxylamine hydrochloride in an acidic medium to form oximes, which exhibited a characteristic ultraviolet absorbance at 233 nm. A standard calibration curve was previously established using glyoxal as the reference standard. The concentration of aldehyde groups was derived from the following linear regression equation:

*X* = ( *Y* – 0.00773 ) / 0.320. (2)

where *Y* represents the ultraviolet absorbance measured at 233 nm and *X* represents the calculated aldehyde concentration. The final mass percentage of aldehyde groups was determined using the following equation:

CHO (%) = ( *X* × 5 × 29 × 0.01 ) / *m* ×100%	(3)

where *X* is the aldehyde concentration derived from the calibration curve and *m* is the dry weight of the sample in g.

**S1.1.3 Molecular weight analysis**

The molecular weight of TFPs and OTFPs was determined in our previous study [1] using gel permeation chromatography under identical sample preparation and analytical conditions. Since the same polysaccharide batch and oxidation protocol were employed, these values are directly applicable to the present work.

**S1.2 Determination of Quercetin (QT) Content**

**S1.2.1 Calibration curve establishment**

The UV-Vis absorption spectra of quercetin (QT) standards were scanned within the release receptor systems consisting of phosphate-buffered saline (PBS, pH 7.4 and 4.5) containing 0.5% Tween 80 to determine the maximum absorption wavelength, which was located at approximately 377 nm. Subsequently, a series of QT standard solutions with gradient concentrations were prepared in the respective pH-specific release media. The absorbance values at 377 nm were recorded to construct the concentration–absorbance calibration curves, and the corresponding linear regression equations were established for subsequent quantitative analysis.

**S1.2.2 Method validation**

To evaluate the specificity of the analytical method, the UV-Vis absorption spectrum of the degradation release medium from the blank TOC hydrogel was scanned under identical testing conditions to determine whether any interfering absorbance existed at 377 nm. The accuracy of the method was validated via spike-recovery assays. Known concentrations of QT standards were introduced into the release media and determined under the same sample pretreatment conditions. The recovery rates were then calculated to thoroughly evaluate the accuracy and reliability of the method.

**S1.2.3 Determination of drug loading**

To evaluate the solubilization behavior and drug loading, excess QT was added to 5% and 10% CMCS solutions and thoroughly mixed under stirring to achieve a state of dispersion equilibrium. After sufficient agitation, the mixtures were centrifuged at 10,000 rpm for 10 min to remove undissolved particles. The supernatants were collected, and the concentration of dissolved QT was determined at 377 nm using the calibration curve established under pH 7.4 conditions.

For the hydrogel samples, the encapsulated drug loading was calculated based on the pre-determined actual QT content within the CMCS precursor solutions. Given that the in situ gelation process proceeded within a completely closed system without any washing steps, significant material loss, or visually detectable precipitation, the total mass of the drug within the system was considered strictly conserved during the gelation process.

**S1.2.4 *In vitro* release studies**

*In vitro* drug release experiments were conducted in PBS (pH 4.5 and 7.4) containing 0.5% Tween 80, utilizing the corresponding pH-specific QT calibration curves. At predefined time intervals, aliquots of the release medium were sampled and centrifuged at 10,000 rpm for 10 min to eliminate potential hydrogel fragments. The resulting supernatants were collected to measure the absorbance at 377 nm, and the released QT concentrations were calculated using the respective standard curves. To eliminate potential matrix interference, all release data were baseline-corrected using the degradation release medium of the blank TOC hydrogel obtained under identical experimental conditions as the background reference.

**S1.3 Water evaporation tests**

Hydrogel samples were prepared as disks with a thickness of 5 mm. Each sample was placed in an open weighing dish and incubated at room temperature. The initial mass was recorded, and the samples were weighed at predetermined time points from 0 to 24 h using an analytical balance. The mass change was used to evaluate water evaporation behavior. Water retention was expressed as the percentage of the remaining mass relative to the initial mass, which was calculated according to the following equation:

Cumulative water loss (%) =((M_0_ - M_t_) / M_0_ × 100%	(4)

where M_0_ is the initial mass of the hydrogel sample at 0 h, and M_t_ is the mass weighed at a predetermined time point t.

**S1.4 Water contact angle measurement**

Water contact angles were measured to evaluate the surface wettability of the hydrogels. Hydrogel samples with a diameter of 20 mm were placed on the sample stage of a contact angle goniometer (OCA20, Dataphysics Instruments GmbH, Germany). A 5 µL droplet of deionized water was gently deposited onto the hydrogel surface using a microsyringe at room temperature. The contact angle was recorded immediately after droplet deposition. The reported values represent the average of three independent measurements taken at different positions on each sample surface.

**S1.5 Morphological Observation**

For TFPs, a 1 wt% TFPs aqueous solution was frozen and lyophilized before imaging. For the TFPs/OTFPs sample, 1 wt% TFPs solution was mixed with 5 wt% OTFPs solution, followed by freezing and lyophilization. For TOC hydrogels, freshly prepared samples were immersed in PBS solutions with different pH values for 20 min, gently blotted to remove excess surface liquid, and then frozen and lyophilized prior to observation. All samples were sputter-coated with gold and imaged using a Hitachi Regulus 8100 SEM.

**S1.6 Cell culture**

HaCaT human keratinocytes were cultured in Dulbecco’s modified Eagle’s medium (DMEM) supplemented with 10% fetal bovine serum (FBS) and 1% penicillin–streptomycin. Cells were maintained at 37 °C in a humidified incubator with 5% CO_2_. The culture medium was replaced every two days, and cells were passaged when reaching approximately 80–90% confluence.

**S1.7 Establishment of a UVB-damaged HaCaT cell model**

HaCaT cells were seeded in 96-well or 24-well plates at appropriate densities and cultured at 37 °C in a humidified atmosphere with 5% CO_2_ for 24 h to allow attachment. The culture medium was then removed, and the cells were gently washed twice with PBS. A sufficient volume of PBS was added to cover the cell layer to prevent drying during UVB exposure. Cells were irradiated with UVB light at a fixed wavelength with the lamp positioned 15 cm above the plate. Irradiation times ranged from 1 to 30 min. Control cells were handled in the same way, except that the plates were wrapped with aluminum foil to block UVB.

After irradiation, PBS was replaced with fresh complete DMEM, and the cells were returned to the incubator for 24 h before further analysis.

Cell viability was determined using the CCK-8 assay. The irradiation time that reduced cell viability to approximately 50% of the untreated control was selected as the UVB damage condition for subsequent experiments.

**S1.8 DCFH-DA fluorescence measurement of quercetin alone**

To evaluate the intrinsic fluorescence of quercetin in the DCFH-DA assay, HaCaT cells were incubated with quercetin in the absence of UVB irradiation. Cells were seeded in 24-well plates at a density of 2 × 10⁴ cells per well and cultured overnight to allow attachment. The medium was then replaced with fresh medium containing quercetin at the same concentration used in the ROS experiments and incubated for 24 h. After incubation, the medium was removed and cells were washed with PBS, and stained following the manufacturer’s protocols. Intracellular ROS levels were evaluated using fluorescence microscopy corresponding treatments. Fluorescence intensity was quantified using ImageJ software. The measured fluorescence signal represents the intrinsic fluorescence of quercetin and was used as a background correction for DCFH-DA measurements in the experimental groups.

**S1.9 Blood Compatibility**

Fresh whole blood was collected from mice and centrifuged at 3000 × g for 15 min. The supernatant was discarded, and the pellet was resuspended in twice the volume of PBS, followed by centrifugation at 3000 × g for 20 min. This washing step was repeated until the upper PBS layer became colorless. Material solutions and distilled water were sterilized by filtration. In 1.5 mL microcentrifuge tubes, 20 μL of prepared blood was mixed with 1 mL of either the material solution, PBS, or ultrapure water. Samples were incubated at 37 °C for 1–2 h, then centrifuged at 3000 × g for 15 min. Photographs of the tubes were taken, and the absorbance of the supernatant was measured at 542 nm using a microplate reader.

**S1.10 Clinical Skin Severity Scoring Criteria**

The clinical skin severity score was graded on a single score from 0 to 4 based on the following criteria:

0 (Normal): Normal skin color; absence of erythema, scaling, or crusting.

1 (Mild): Slight erythema, minor roughness, or punctate crusting without erosion.

2 (Moderate): Definite erythema/edema, focal scaling, or small crusts (<50% of the damaged area).

3 (Severe): Extensive crusting, localized erosion, or severe scaling (≥50% of the damaged area).

4 (Very severe): Widespread tissue necrosis, deep ulceration, or severe hemorrhage.

**S1.11 Skin Patch Safety**

The hydrogel was applied to the dorsal skin of guinea pigs once daily for 7 consecutive days. Skin reactions were recorded daily.

**S2. Results**

**Figure S1.** Method validation for quercetin (QT) quantification. (A) UV-Vis spectra (300–500 nm) of QT standard and blank hydrogel release medium under pH 7.4 (left) and pH 4.5 (right) conditions. (B) Representative UV–Vis calibration curves of QT in pH 7.4 (left) and pH 4.5 (right) PBS containing 0.5% Tween 80. The calibration curves were used for analytical quantification of QT in release studies.

**Figure S2.** SEM images of TFPs and TFPs/OTFPs Composite. (A–C) TFPs, (D–F) TFPs/OTFPs

**Figure S3.** Cumulative water loss of different hydrogel samples over time.

**Figure S4.** Water Contact Angle Measurements of Three Hydrogel Samples. (A) GC, (B) OC, (C) TOC

**Figure S5.** UV absorption of hydrogels.

A. 200-500nm, B. 320-400nm, C. 280-320nm.

**Figure S6.** SEM images of TOC hydrogel equilibrated in PBS at (A) pH 4.5 and (B) pH 7.4. Scale bar = 200 µm; magnification = 50×.

**Figure S7.** Korsmeyer–Peppas kinetic fitting curves of quercetin release from the hydrogels in different pH media (pH 7.4 and pH 4.5) within the initial 12 h.

**Figure S8.** Representative image of DCFH-DA incubated with QT alone (without UVB)

**Figure S9.** Representative hemolysis tubes and quantified hemolysis (%). (A) TOC-QT. (B) TOC. Data are presented as mean ± SD (n = 3).

**Figure S10.** Dorsal skin after daily topical application for 7 days.

**Figure S11.** Survey XPS spectra of CMCS and TOC hydrogel. A Cl 2p signal observed in the TOC spectrum originates from residual chloride introduced during pH adjustment with HCl and is not related to hydrogel crosslinking.

| **Medium** | **Added (µg/mL)** | **Recovery Range (%)** |
| --- | --- | --- |
| pH 7.4 PBS 0.5% Tween 80 | 5 | 94.71 ~ 101.83 |
|  | 10 | 95.13 ~ 97.80 |
|  | 15 | 94.97 ~ 96.65 |
| pH 4.5 PBS 0.5% Tween 80 | 5 | 97.29 ~ 100.46 |
|  | 10 | 96.33 ~ 97.63 |
|  | 15 | 94.96 ~ 96.59 |

**Table S1.** Recovery of QT in release media (n = 3).

**S2.1 Validation of the UV-Vis Analytical Method**

The UV-Vis spectrophotometric method was validated for its specificity and accuracy. To evaluate specificity, the full UV-Vis spectra of the blank TOC hydrogel release medium and the QT standard were compared. The blank hydrogel medium showed no absorption at 377 nm under both pH 7.4 and pH 4.5 conditions. This result proves that the hydrogel matrix does not interfere with QT quantification.

The accuracy of the method was verified using spike-recovery tests at three concentration levels (5, 10, and 15 µg/mL). As summarized in Table S1, the recovery ranges for QT were 94.71% ~ 101.83% in pH 7.4 medium and 94.96% ~ 100.46% in pH 4.5 medium. These recovery values meet the standard criteria for method validation. Therefore, this UV-Vis method is suitable for monitoring QT loading and release behaviors.

**S2.2 FTIR analysis of the hydrogels**

FTIR spectra are presented in Figure 1A. Only the OTFP/TFPs sample showed an aldehyde-related band at 2847 cm^-1^, indicating the presence of oxidized polysaccharide. Apart from this feature, the spectra were largely similar to that of TFPs, suggesting that simple mixing did not introduce new covalent bonds. A band at 1318 cm^-1^, attributed to C–N stretching and O–H bending in the CMCS backbone, appeared in CMCS and in all three hydrogels. All samples exhibited a broad O–H band centered near 3280 cm^-1^, which became broader in the hydrogels compared with the precursors, indicating stronger hydrogen bonding. In the COO⁻/C=N region, the precursor peaks at 1582 cm^-1^ (CMCS) and 1598 cm^-1^ (TFPs) shifted and merged at approximately 1590 cm^-1^ in the hydrogels, consistent with the formation of Schiff-base crosslinks. The band at 1410 cm^-1^ (COO^-^ symmetric stretching) showed little shift among the samples. The characteristic polysaccharide bands at 2916 cm^-1^ (C–H stretching) and 1020 cm^-1^ (C–O–C vibration) were preserved in all spectra, indicating that the main polysaccharide backbone remained intact.

**S2.3 Water evaporation and water contact angle**

Water evaporation and static water contact angles were measured for the three hydrogels (Figures S3 and S4). GC exhibited the lowest evaporation rate. This behavior is consistent with its dense chemically crosslinked network, which restricts water mobility. The consumption of hydroxyl groups during crosslinking also resulted in the largest contact angle. OC showed the fastest evaporation rate, which reflects its relatively loose network structure. The surface retained more hydrophilic groups, giving an intermediate contact angle. TOC formed a semi-IPN structure in which the chemically crosslinked network was combined with physically entangled polysaccharide chains. This structure slowed water diffusion and reduced evaporation. At the same time, more free hydroxyl groups remained on the surface, resulting in the lowest contact angle.

**S2.4 DCFH-DA assay with quercetin alone without UVB exposure**

Because quercetin has intrinsic fluorescence that can interfere with DCFH-DA detection, a control experiment was performed by incubating HaCaT cells with quercetin alone in the absence of UVB irradiation. A measurable fluorescence signal was observed due to the intrinsic fluorescence of quercetin. Quantitative analysis showed that the fluorescence intensity of quercetin alone was 13.94 ± 1.87 (n = 3). Therefore, the corresponding quercetin-derived fluorescence was subtracted from the DCFH-DA measurements in the experimental groups to correct for this background signal.

**S2.5 Blood Compatibility**

The blood compatibility and skin tolerance of the hydrogel were evaluated through *in vitro* and *in vivo* assays using whole blood from BALB/c mice and repeated topical application, respectively. In the hemolysis assay, deionized water (ddH₂O) and PBS served as positive and negative controls. Treatment with TOC-QT at 10, 5, and 1 mg/mL did not induce significant hemolysis, with hemolysis rates below 3% (Figure S9), substantially lower than the positive control, indicating favorable blood compatibility *in vitro*.

**S2.6 Skin Patch Safety**

For the repeated skin exposure test, the hydrogel was applied daily to the dorsal skin of mice for 7 consecutive days, and skin responses were monitored (**Figure S10**). No obvious erythema, erosion, exudation, or alterations in skin color and morphology were observed in any group throughout the observation period.

Overall, the natural semi-IPN hydrogel exhibited good *in vitro* blood compatibility and short-term skin tolerance, supporting its preliminary biocompatibility as a topical delivery system and its potential for further preclinical evaluation.

**References**

[1] F. Lin, Z. Wu, F. Luo, Z. Lin, J. Wang, W. Wang, R. Li, G. Fang, B. Qiu, H_2_O_2_-driven dual-functional modification of Tremella fuciformis polysaccharides: molecular weight reduction and oxidative group engineering for multidimensional bioactivity control, Int. J. Biol. Macromol., 331 (2025) 148513. <https://doi.org/10.1016/j.ijbiomac.2025.148513>.
